# E3 ubiquitin ligase UBR5 promotes gemcitabine resistance in pancreatic cancer by inducing O-GlcNAcylation-mediated EMT via destabilization of OGA

**DOI:** 10.1038/s41419-024-06729-z

**Published:** 2024-05-16

**Authors:** Yunyan Du, Zhangjian Yang, Hao Shi, Zhihan Chen, Rong Chen, Fan Zhou, Xiaogang Peng, Tao Hong, Liping Jiang

**Affiliations:** 1 School of Pharmacy, Jiangxi Medical College, Nanchang University, Nanchang, 330006 China; 2 Key Laboratory of Drug Targets and Drug Screening of Jiangxi Province, Nanchang University, Nanchang, 330006 China; 3 Department of General Surgery, The Second Affiliated Hospital of Nanchang University, Nanchang, 330006 China; 4 Jiangxi Province Key Laboratory of Molecular Medicine, The Second Affiliated Hospital of Nanchang University, Nanchang, 330000 China; 5 Department of Neurosurgery, The First Affiliated Hospital of Nanchang University, Nanchang, 330006 China

**Keywords:** Pancreatic cancer, Oncogenes

## Abstract

Pancreatic cancer (PC) is among the deadliest malignancies, with an extremely poor diagnosis and prognosis. Gemcitabine (GEM) remains the first-line drug for treating PC; however, only a small percentage of patients benefit from current immunotherapies or targeted therapies. Resistance to GEM is prevalent and affects long-term survival. We found that ubiquitin-protein ligase E3 module N-recognition 5 (*UBR5*) is a therapeutic target against GEM resistance. *UBR5* was markedly upregulated in clinical GEM-resistant PC samples and GEM-resistant PC cells. *UBR5* knockdown markedly increased GEM sensitivity in GEM-resistant PC cell lines. UBR5-mediated GEM resistance was accompanied by activation of epithelial-mesenchymal transition (EMT) and could be mitigated by inhibiting EMT. Further analysis revealed that UBR5 promoted GEM resistance in PC cells by enhancing O-GlcNAcylation-mediated EMT. In addition, UBR5 knockdown resulted in increased O-GlcNAase (OGA) levels, an essential negatively regulated enzyme in the O-GlcNAcylation process. We identified a negative association between OGA and UBR5 levels, which further supported the hypothesis that O-GlcNAcylation-mediated GEM resistance induced by UBR5 is OGA-dependent in PC cells. Mechanistic studies revealed that UBR5 acts as an E3 ubiquitin ligase of OGA and regulates O-GlcNAcylation by binding and modulating OGA, facilitating its degradation and ubiquitination. Additionally, high-throughput compound library screening using three-dimensional protein structure analysis and drug screening identified a Food and Drug Administration drug, Y-39983 dihydrochloride, as a potent GEM sensitiser and UBR5 inhibitor. The combination of Y-39983 dihydrochloride and GEM attenuated tumour growth in a mouse xenograft tumour model. Collectively, these data demonstrated that UBR5 plays a pivotal role in the sensitisation of PC to GEM and provides a potential therapeutic strategy to overcome GEM resistance.

## Background

Pancreatic cancer (PC) is among the deadliest diseases in the developed world owing to its late diagnosis [1]. PC has a 5-year survival rate of < 5% and is projected to be the second most common cause of cancer-related deaths by 2030 [2]. Most patients with PC do not recognise their worsening condition until they progress to advanced disease stages [3]. The primary reason for low survival rates is attributed to initial diagnosis of early local infiltration and distant metastases [1, 2]. Poor response to chemotherapy and resistance in PC remains a major clinical challenge, yielding poor overall prognosis [4]. Gemcitabine (GEM), a deoxycytidine analogue that suppresses DNA replication and tumour development, is a single chemotherapeutic agent extensively employed to treat PC [5]. GEM-based chemotherapy regimens are used in patients who are particularly unresponsive to other therapies [5]. However, many patients with PC rapidly develop GEM resistance, dramatically hindering their survival [6]. Hence, identifying GEM resistance mechanisms and drug combinations that improve GEM efficacy is urgently needed.

Cancer cells typically undergo remodelling of their energy metabolism [7]. A newly emerging mechanism underlying this process under glucose metabolism is O-GlcNAcylation, an atypical glycosylation pathway activated in response to stimuli such as cellular stress and nutrient deprivation [8]. O-GlcNAcylation is an essential mode of the post-translational modification of substrate proteins [9]. UDP-GlcNAc transfers O-conjugated-β-N-acetylglucosamine (O-GlcNAc) to O-GlcNAc transferase (OGT) [10]. This enzyme links O-GlcNAc molecules to the serine and threonine residues of substrate proteins and those encompassing mitochondrial, nuclear, and cytoplasmic proteins [11]. Subsequently, O-GlcNAase (OGA) reverses this process by hydrolysing OGT [12]. Unlike other post-translational modifications (PTM), O-GlcNAcylation is strictly regulated by OGA and OGT [13]. O-GlcNAcylation is crucial for the development of malignant tumours and drug resistance [14, 15]. In lung cancer cells, hyper-O-GlcNAcylation is linked to cisplatin resistance [16]. Thus, further research is required to clarify the function of O-GlcNAcylation in drug resistance and identify molecules that target hyper-O-GlcNAcylation.

Human ubiquitin-protein ligase E3 module N-recognition 5 (UBR5, or EDD) contains a structural domain homologous to the E6-AP carboxyl terminus (HECT) and was originally identified as a luteinising hormone regulatory gene in breast cancer cells [17, 18]. UBR5 belongs to the E6-AP carboxy-terminal family and targets specific proteins involved in ubiquitin-induced proteolysis [19]. UBR5 influences transcription mechanisms, cell cycle, DNA damage response, apoptosis, and metabolism [20, 21]. Furthermore, UBR5 functions as an oncogene and is highly expressed in various cancerous tissues [17, 22], particularly in gastric, breast, gallbladder, lymphoma, and ovarian cancer cells [17, 19, 22]. We previously demonstrated that patients with PC and high UBR5 levels exhibit poorer prognoses, and UBR5 was abundantly expressed in PC [23]. Additionally, UBR5 is essential for tumour chemoresistance, and *UBR5* overexpression leads to cisplatin resistance in ovarian cancer cell lines [24]. However, the function and role of UBR5 in GEM resistance remain unclear.

This study investigated the role of UBR5 in dysregulating O-GlcNAcylation-mediated epithelial-mesenchymal transition (EMT) and conferring GEM resistance in PC and clarified the underlying molecular mechanisms. Furthermore, this study offers preclinical evidence of the therapeutic potential of inhibiting UBR5 in chemotherapy-resistant PC. We extensively characterised the inhibitory effect of a Food and Drug Administration (FDA) drug, Y-39983 dihydrochloride, on GEM resistance in PC in vitro and in vivo. These findings highlight the potential of Y-39983 dihydrochloride combination therapies, which should be evaluated and optimised in clinical trials.

## Results

### UBR5 is highly expressed in GEM-resistant pancreatic cancer tissues and cells

**Fig. 1:**
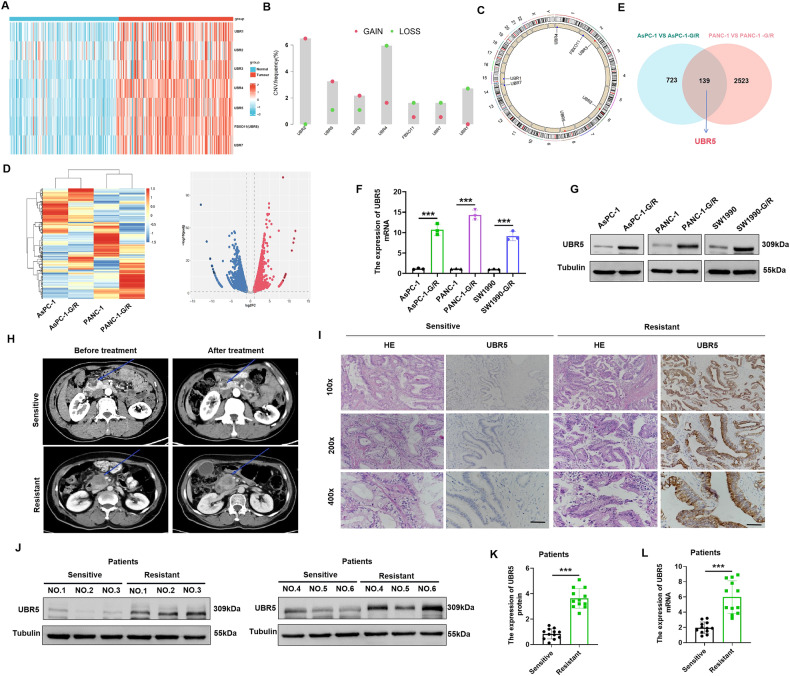
UBR5 is highly expressed in gemcitabine-resistant pancreatic cancer tissues. **A** Differential expression of the *UBR* genes in pancreatic cancer and adjacent tissues. **B**, **C** Genomic characteristics of *UBR* genes in pancreatic cancer. **B** Distribution of copy number variants. **C** Genome location. **D** Heat map displaying the microarray data of parental pancreatic cancer cells and gemcitabine-resistant pancreatic cancer cells. **E** Intersection of genes with increased expression in gemcitabine-resistant AsPC-1 and PANC-1 cells. **F** Quantitative reverse transcription-polymerase chain reaction analysis of *UBR5* mRNA expression in parental pancreatic cancer and gemcitabine-resistant cells (***P < 0.001). **G** Western blot analyses of UBR5 expression in parental pancreatic cancer and gemcitabine-resistant cells. **H** Computed tomography imaging of pancreatic cancer patients before and after treatment. **I** Representative immunohistochemical staining of UBR5 in gemcitabine-sensitive and gemcitabine-resistant tissues of pancreatic cancer. **J**, **K** Western blot examination of UBR5 expression in gemcitabine-sensitive and gemcitabine-resistant tissues of pancreatic cancer (***P < 0.001). **L** qRT-PCR analysis of UBR5 expression in gemcitabine-sensitive and gemcitabine-resistant tissues of pancreatic cancer (***P < 0.001).

The expression of UBR box E3 ligases (UBRs) is associated with the malignant development of tumours and their drug resistance [25]. The expression of seven UBRs was significantly higher in tumour tissues than in adjacent tissue samples (Fig. 1A). Investigation of the frequency of copy number variation (CNV) alterations revealed a prevalence in the UBRs; UBR2, UBR3, and UBR5 presented more copy number amplifications, and UBR1, UBR4, UBR6, and UBR7 presented more copy numbers (Fig. 1B). The locations of CNV alterations in UBRs on the chromosomes are shown in Fig. 1C. Subsequently, we have generated a GEM-resistant PC cell line in a previous study [26]. To determine the gene set that may influence the sensitivity of PC cells to chemotherapy, gene expression in parental PANC-1, AsPC-1, PANC-1-G/R, and AsPC-1-G/R cells was compared using RNA-seq. The difference in gene expression between resistant and parental cells is shown in the volcano plot (Fig. 1D). UBR5 was found to be remarkably higher in GEM-resistant cells than in the parental cells (Fig. 1E). Furthermore, *UBR5* expression levels in AsPC-1-G/R, PANC-1-G/R, and SW1990-G/R cells were analysed by qRT-PCR. *UBR5* mRNA expression was higher in the GEM-resistant PC cell lines compared with that in the parental PC cell lines (Fig. 1F). UBR5 protein expression was consistently high in GEM-resistant PC cell lines (Fig. 1G). These findings indicate that UBR5 may confer GEM resistance to PC cells. We then examined UBR5 expression in GEM-resistant and GEM-sensitive PC tissues. Immunohistochemistry analyses (IHC) revealed that UBR5 expression in GEM-resistant PC tissues was markedly upregulated compared with that in GEM-sensitive PC tissues (Fig. 1H, I). UBR5 expression was markedly increased in GEM-resistant PC tissues (Fig. 1J–L). These results indicate that PC tissues and GEM-resistant cells have high levels of UBR5, which is associated with GEM-resistance.

### Suppression of UBR5 raises the chemotherapeutic sensitivity of PC to GEM in vitro and in vivo

**Fig. 2:**
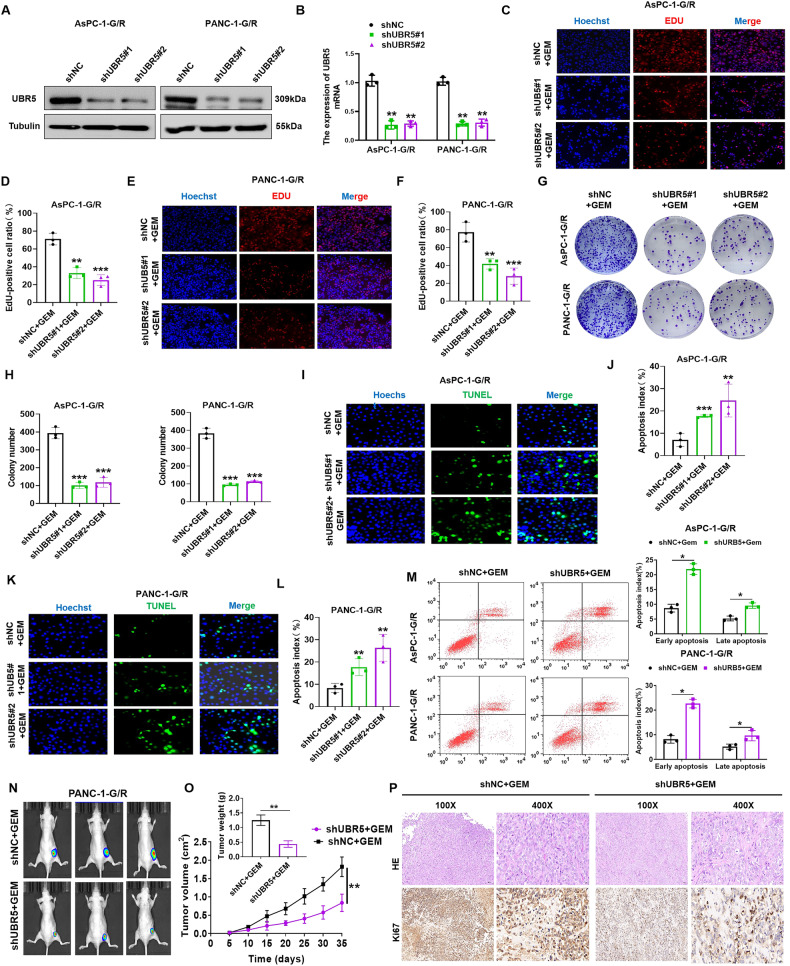
Knockdown of UBR5 increases pancreatic cancer sensitivity to gemcitabine in vivo and in vitro. **A** Western blot analysis of UBR5 expression in shUBR5-AsPC-1-G/R and shUBR5-PANC-1-G/R cells. **B** qRT-PCR analysis of UBR5 expression in shUBR5-AsPC-1-G/R and shUBR5-PANC-1-G/R cells. **C**, **D** Quantification and representative images of EdU assays for AsPC-1-G/R cells transfected with shUBR5 plasmids (**P < 0.01, ***P < 0.001). **E**, **F** Quantification and representative images of EdU assays for PANC-1-G/R cells transfected with shUBR5 plasmids (**P < 0.01, ***P < 0.001). **G**, **H** Quantification and representative images of colony formation assays for AsPC-1-G/R or PANC-1-G/R cells transfected with shUBR5 plasmids (***P < 0.001). **I**, **J** Quantification and representative images of TUNEL assays for the AsPC-1-G/R cells transfected with shUBR5 plasmids (**P < 0.01, ***P < 0.001). **K**, **L** Quantification and representative images of TUNEL assays for PANC-1-G/R cells transfected with shUBR5 plasmids (**P < 0.01). **M** Results are expressed as a scatter diagram for the measurement of apoptotic cells and as a calculated percentage of the annexin-V-positive cell population in shUBR5-AsPC-1-G/R or shUBR5-PANC-1-G/R cells (**P < 0.05). **N**, **O** shUBR5/PANC-1-G/R cells were subcutaneously injected into nude mice, and the tumour volumes were detected on the indicated dates; at the end of the experiment, tumours were dissected, weighed, and imaged. (**P < 0.01). **P** Representative hematoxylin and eosin and immunohistochemical staining of Ki67 in tumour tissues isolated from different nude mice groups.

We explored whether decreasing UBR5 levels made PC more sensitive to GEM. We first stably transfected two UBR5-specific shRNAs (shUBR5-) into AsPC-1-G/R and PANC-1-G/R cells. shUBR5#1 and shUBR5#2 significantly reduced UBR5 expression in stable cell lines compared to scrambled shRNA (Fig. 2A, B). 5-Ethynyl-2’-deoxyuridine (EdU) and Colony formation assays were performed to assess cell proliferation and viability. Decreasing UBR5 expression increased the inhibitory effect of GEM on AsPC-1-G/R and PANC-1-G/R cell proliferation (Fig. 2C–H). We performed terminal deoxynucleotidyl transferase dUTP nick end labeling (TUNEL) and flow cytometry assays to assess the effect of UBR5 knockdown on the sensitivity of AsPC-1-G/R and PANC-1-G/R cells to GEM. UBR5 knockdown increased the apoptotic rate of AsPC-1-G/R and PANC-1-G/R cells in response to GEM (Fig. 2I–M). These findings suggest that *UBR5* knockdown increases the sensitivity of PC cells to GEM in vitro.

Next, the effect of UBR5 on GEM resistance was assessed in vivo using a xenograft tumour mouse model. GEM was administered to nude mice after injecting sh-UBR5-PANC-1-G/R and sh-NC-PANC-1-G/R cells. Tumour weight and volume were markedly reduced in the sh-UBR5-PANC-1-G/R groups compared with those in the sh-NC-PANC-1-G/R groups (Fig. 2N, O). The IHC assay showed that the sh-UBR5-PANC-1-G/R group exhibited substantially lower cell proliferation rates (Ki67) (Fig. 2P). These findings demonstrate that suppression of UBR5 increases the sensitivity of PC to GEM in vitro and in vivo.

### UBR5 promotes GEM resistance by inducing EMT

**Fig. 3:**
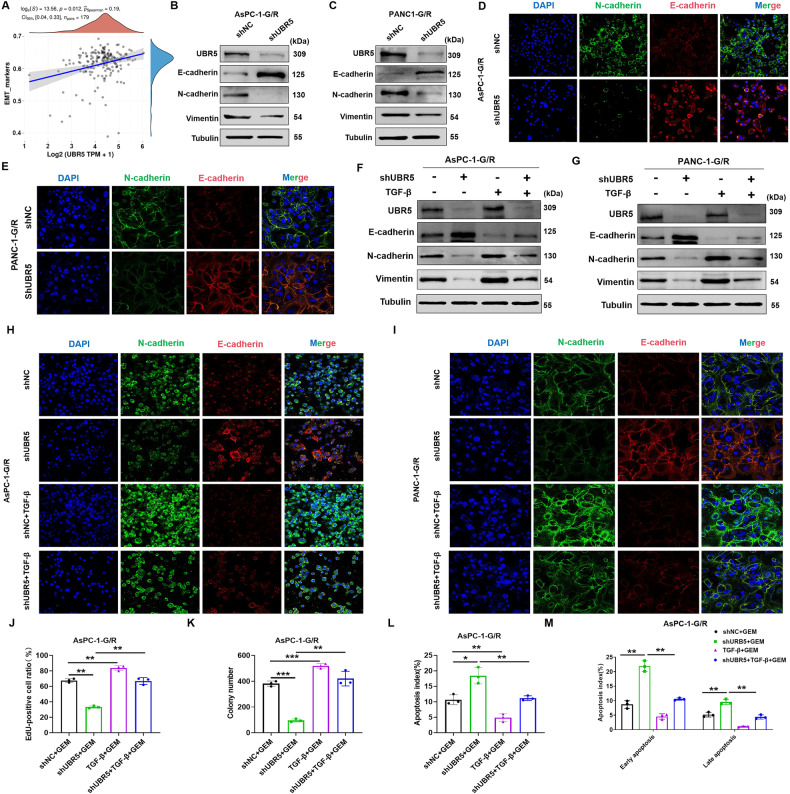
UBR5 promotes EMT to enhance gemcitabine resistance in pancreatic cancer cells. **A** Spearman correlation analysis of the correlation between UBR5 and the EMT pathway score. UBR5 expression is represented by the abscissa, and the EMT pathway score is represented by the ordinate. A density curve to the right represents the trend in the distribution of pathway scores, a density curve to the upper part represents the trend in the distribution of gene expression. The top part shows the *p*-value, correlation coefficient, and correlation calculation method. **B**, **C** Western blot analysis of the effect of inhibiting UBR5 on the expression of EMT-related proteins (E-cadherin, N-cadherin, and vimentin) in AsPC-1-G/R and PANC-1-G/R cells. **D**, **E** Immunofluorescence analysis of the effect of inhibiting UBR5 on the expression of EMT-related proteins (E-cadherin and N-cadherin) in AsPC-1-G/R and PANC-1-G/R cells. **F**, **G** Western blot analysis was used to observe the expression of EMT-related proteins in the indicated treatment group. **H**, **I** Immunofluorescence analysis was used to observe the expression of EMT-related proteins (E-cadherin and N-cadherin) in the indicated treatment group. **J** Cell viability was detected using EdU assay of AsPC-1-G/R cells subjected to the indicated treatments (**P < 0.01). **K** Cell viability was detected using a colony formation assay of AsPC-1-G/R cells subjected to the indicated treatments (**P < 0.01, ***P < 0.001). **L**, **M** The TUNEL or flow cytometry assay to examine apoptosis in AsPC-1-G/R cells subjected to the indicated treatments (*P < 0.05, **P < 0.01).

We utilised the Cancer Genome Atlas (TCGA) database to gather RNA sequencing data together with relevant clinical information from 179 patients with PC. Single-sample Gene Set Enrichment Analysis (ssGSEA) revealed a strong association between the EMT pathway and UBR5 expression (*p* = 0.012, Fig. 3A). Previous studies have confirmed that EMT causes drug resistance in many solid tumours, particularly PC [27]. Thus, we speculated that UBR5 may lead to GEM resistance in PC cells by promoting EMT. To confirm this, we downregulated UBR5 expression in GEM-resistant PC cells and performed western blot assays to track changes in the expression of EMT markers (E-cadherin, N-cadherin, and vimentin). When UBR5 expression decreased, E-cadherin expression increased while N-cadherin and vimentin decreased, suggesting that decreased UBR5 expression can prevent EMT in GEM-resistant PC cells (Fig. 3B, C). Furthermore, an immunofluorescence analyisis was performed to observe changes in the expression of EMT markers following reduced UBR5 expression. Reducing UBR5 expression resulted in increased E-cadherin and decreased N-cadherin expression (Fig. 3D, E). To verify that UBR5 influences PC cell resistance through EMT, we lowered UBR5 expression in PC cells resistant to GEM and introduced the EMT activator Transforming Growth Factor-β (TGF-β) to track alterations in GEM sensitivity in AsPC-1-G/R and PANC-1-G/R cells. Reducing UBR5 expression increased the sensitivity of AsPC-1-G/R and PANC-1-G/R cells; however, incorporating EMT activators prevented this process (Fig. 3F, M and Supplementary Fig. 1A–D). Therefore, UBR5 promotes GEM resistance in PC by promoting EMT.

### UBR5 promotes GEM resistance in PC by enhancing O-GlcNAcylation-mediated EMT

**Fig. 4:**
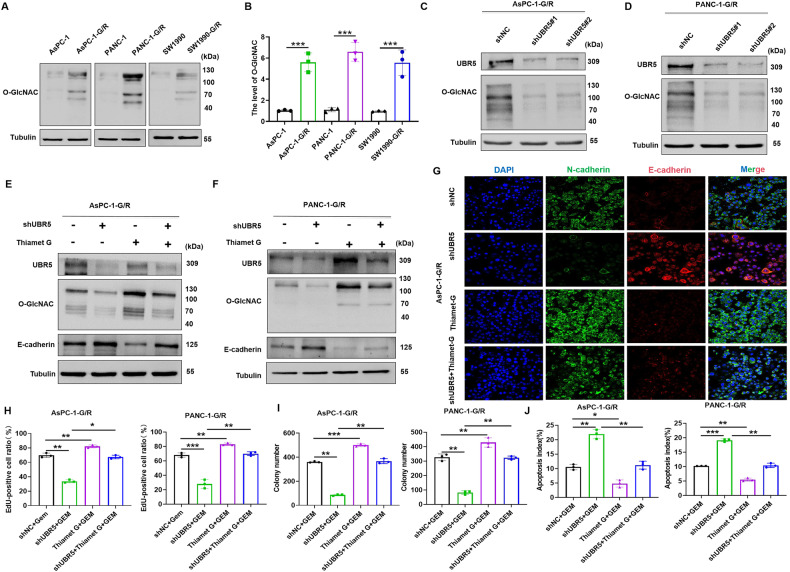
UBR5 regulation of O-GlcNAcylation-mediated EMT leads to gemcitabine resistance in pancreatic cancer. **A**, **B** Western blot analyses of O-GlcNAcylation levels in parental pancreatic cancer and gemcitabine-resistant cells (***P < 0.001). **C**, **D** Western blot analysis of O-GlcNAcylation levels in shUBR5-AsPC-1-G/R and shUBR5-PANC-1-G/R cells. **E**, **F** Western blot analysis of UBR5, E-cadherin expression, and O-GlcNAcylation levels in AsPC-1-G/R and PANC-1-G/R cells subjected to the indicated treatments. **G** Immunofluorescence analysis was used to observe the expression of EMT-related proteins (E-cadherin and N-cadherin) in indicated treatment group. **H** Cell viability was detected using the EdU assay of AsPC-1-G/R and PANC-1-G/R cells subjected to the indicated treatments (**P < 0.05, **P < 0.01). **I** Cell viability was detected using a colony formation assay of AsPC-1-G/R and PANC-1-G/R cells subjected to the indicated treatments (**P < 0.01, ***P < 0.001). **J** The TUNEL assay to examine apoptosis in AsPC-1-G/R and PANC-1-G/R cells subjected to the indicated treatments (*P < 0.05, **P < 0.01, ***P < 0.001).

O-GlcNAcylation is an important post-translational modification that affects chemotherapeutic sensitivity via EMT [28]. Therefore, we speculated that UBR5 promotes GEM resistance in PC by enhancing O-GlcNAcylation-mediated EMT. First, O-GlcNAcylation levels in AsPC-1-G/R, PANC-1-G/R, and SW1990-G/R cells were analysed by western blot. The levels of O-GlcNAcylation were elevated in GEM-resistant cells compared to those in the parental PC cell lines (Fig. 4A, B). We then measured the O-GlcNAcylation levels in GEM-resistant PC cells with UBR5 knockdown to further identify whether UBR5 could modulate O-GlcNAcylation. PANC-1-G/R and AsPC-1-G/R cells had considerably low O-GlcNAcylation levels following UBR5 knockdown (Fig. 4C, D), implying that O-GlcNAcylation is modulated by UBR5. Furthermore, Thiamet G, a glycosyl agonist, was used to increase O-GlcNAcylation levels in UBR5-knockdown GEM-resistant cells. We demonstrated that UBR5 controls O-GlcNAcylation levels and causes EMT and GEM resistance. Using immunofluorescence analysis and western blotting, we assessed the expression of UBR5, EMT markers, O-GlcNAcylation, and cell proliferation. Downregulation of UBR5 lowered O-GlcNAcylation. Furthermore, increased O-GlcNAcylation attenuated the loss of N-cadherin in UBR5-knockdown PANC-1-G/R and AsPC-1-G/R cells (Fig. 4E–G and Supplementary Fig. 2). Moreover, UBR5 knockdown significantly decreased GEM-resistant PC cell proliferation, whereas increased O-GlcNAcylation rescued cell proliferation in UBR5 knockdown cells (Fig. 4H, I). In addition, TUNEL findings demonstrated that following GEM treatment, apoptosis in shUBR5-transfected cells was reduced by elevated O-GlcNAcylation levels (Fig. 4J).

Next, OSMI-1, a glycosyl inhibitor, was used to decrease O-GlcNAcylation levels in flag-UBR5 GEM-resistant cells. Western blot results revealed that overexpression of UBR5 significantly decreased E-cadherin expression, whereas OSMI-1 dramatically inhibited the decrease of E-cadherin expression induced by UBR5 in PANC-1-G/R cells (Supplementary Fig. 3A). Meanwhile, UBR5 significantly increased GEM-resistant PC cell proliferation, while decreasing O-GlcNAcylation inhibited cell proliferation in flag-UBR5 GEM-resistant cells (Supplementary Fig. 3B, C). Moreover, TUNEL findings demonstrated that following GEM treatment, apoptosis in flag-UBR5-transfected cells was increased by inhibiting O-GlcNAcylation levels (Supplementary Fig. 3D). These data suggest that in GEM-resistant PC cells, O-GlcNAcylation is essential for UBR5 to induce GEM resistance and EMT.

### UBR5 promotes GEM resistance by inducing O-GlcNAcylation-mediated EMT via OGA

**Fig. 5:**
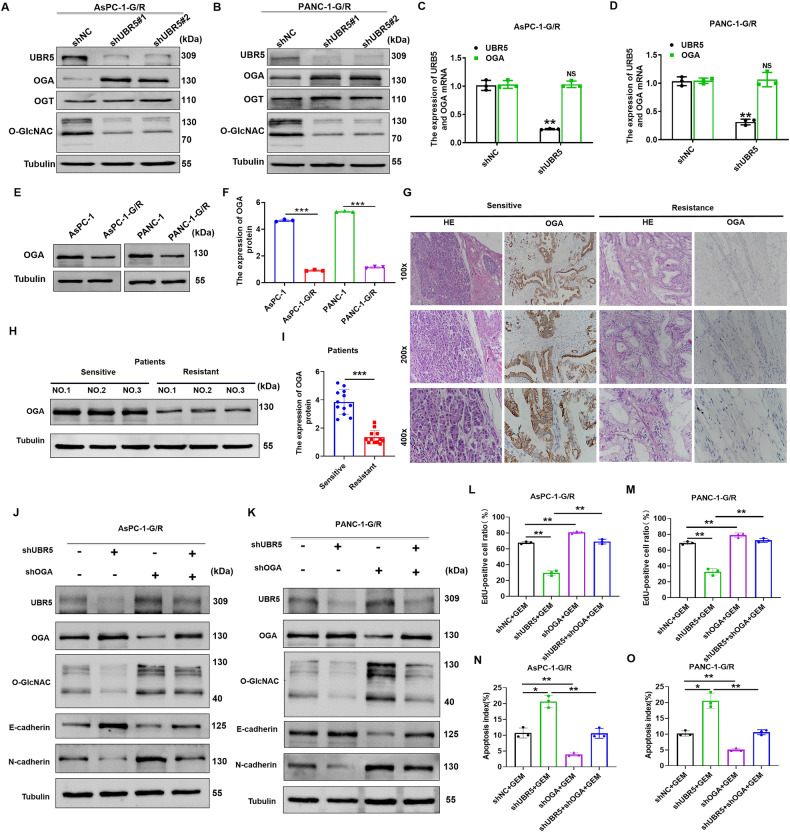
UBR5 regulates O-GlcNAcylation-mediated EMT by inactivating OGA in pancreatic cancer cells. **A**, **B** Western blot analysis of UBR5, OGA, OGT expression, and O-GlcNAcylation levels in shUBR5-AsPC-1-G/R and shUBR5-PANC-1-G/R cells. **C**, **D** qRT-PCR analysis of OGA expression in shUBR5-AsPC-1-G/R and shUBR5-PANC-1-G/R cells. **E**, **F** Western blot analyses of UBR5 expression in pancreatic cancer parental and gemcitabine-resistant cells (***P < 0.001). **G** Representative immunohistochemical staining of OGA in gemcitabine-sensitive and gemcitabine-resistant pancreatic cancer tissues. **H**, **I** Western blot examination of OGA expression in gemcitabine-sensitive and gemcitabine-resistant pancreatic cancer tissues (***P < 0.001). **J**, **K** Western blot analysis of UBR5, OGA, E-cadherin, N-cadherin expression, and O-GlcNAcylation levels in AsPC-1-G/R and PANC-1-G/R cells subjected to the indicated treatments. **L**, **M** Cell viability was detected using the EdU assay of AsPC-1-G/R and PANC-1-G/R cells subjected to the indicated treatments (**P < 0.01). **N**, **O** The TUNEL assay to examine apoptosis in AsPC-1-G/R and PANC-1-G/R cells subjected to the indicated treatments (*P < 0.05, **P < 0.01).

Unlike other PTMs, O-GlcNAcylation is tightly controlled by OGT and OGA [13]. We initially examined OGA and OGT expression in UBR5-knockdown GEM-resistant PC cell lines to better understand how UBR5 modulates O-GlcNAcylation and affects GEM sensitivity. UBR5 knockdown dramatically boosted OGA expression in AsPC-1-G/R and PANC-1-G/R cells, but did not change OGT protein expression (Fig. 5A, B). OGA and OGT mRNA levels were unchanged after reducing UBR5 expression in AsPC-1-G/R and PANC-1-G/R cells (Fig. 5C, D). The expression levels of OGA protein in the GEM-resistant PC cell lines were lower than those in the parental cell lines (Fig. 5E, F). A notable decrease in OGA protein expression was observed when comparing GEM-resistant to GEM-sensitive PC tissues (Fig. 5G–I). Scatter plots revealed that UBR5 and OGA protein expression levels were negatively correlated in PC tissues (Supplementary Fig. 4). These results indicate that UBR5 regulates O-GlcNAcylation levels via OGA, thereby promoting EMT and leading to GEM resistance in PC cells. Furthermore, we examined the expression of UBR5, EMT markers, O-GlcNAcylation, OGA, and cell proliferation after silencing OGA expression in UBR5-knockdown GEM-resistant PC cells. The results showed that UBR5 downregulation increased OGA protein expression, whereas OGA downregulation attenuated the loss of N-cadherin expression in UBR5-knockdown AsPC-1-G/R and UBR5-knockdown PANC-1-G/R cells (Fig. 5J, K). Moreover, downregulation of OGA inhibited the decrease in O-GlcNAcylation levels, cell proliferation, and EMT observed in UBR5-knockdown GEM-resistant PC cells (Fig. 5J–M). In addition, TUNEL assay results showed that OGA silencing reduced apoptosis in shUBR5-transfected cells after treatment with GEM (Fig. 5N, O). These findings revealed that UBR5 promotes GEM resistance by inducing O-GlcNAcylation-mediated EMT depend on OGA.

### UBR5 destabilizes OGA by modulating OGA ubiquitination in PC cells

**Fig. 6:**
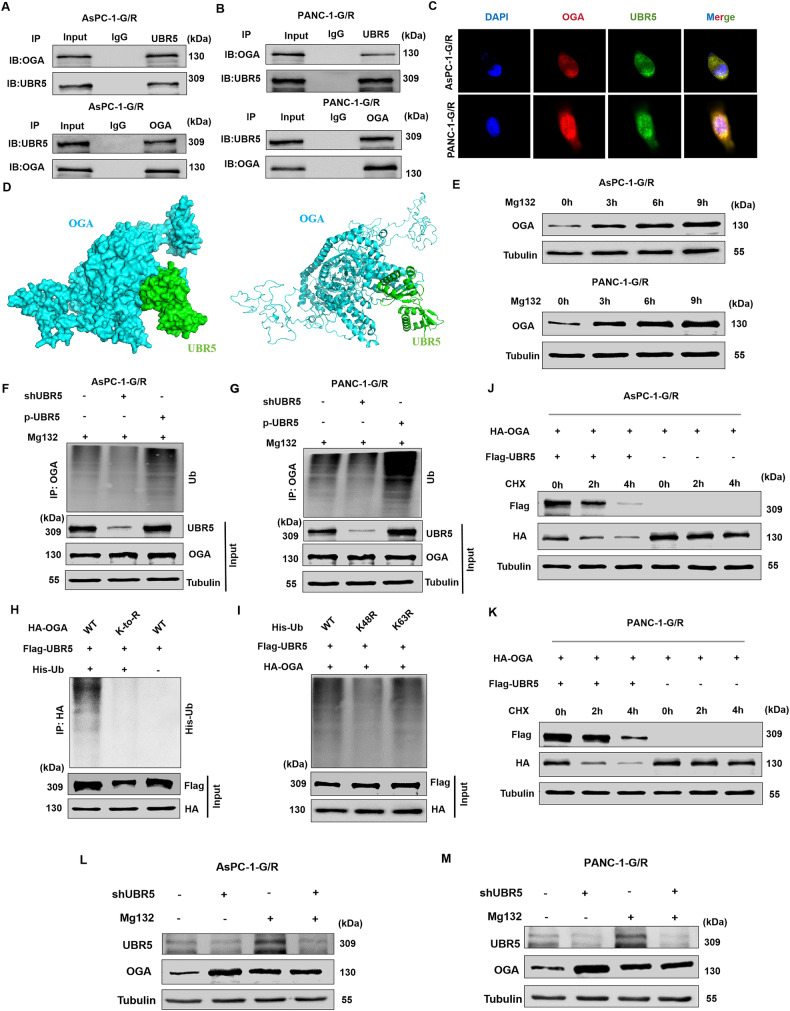
UBR5 destabilises OGA by regulating the ubiquitination of OGA in pancreatic cancer cells. **A**, **B** Co-immunoprecipitation (Co-IP) for UBR5 and OGA in AsPC-1-G/R and PANC-1-G/R cells. **C** Co-localisation studies of pancreatic cancer cells using anti-UBR5 antibody (1:100, green) and anti-OGA antibody (1:100, red), followed by DAPI nuclear counterstaining (blue). The merged images of UBR5 (green) and OGA (red) with DAPI (blue) are also shown. **D** Docking analysis results for the binding of UBR5 and OGA. **E** AsPC-1-G/R and PANC-1-G/R cells were treated with MG132 for the indicated times, and levels of OGA were determined. **F**, **G** Knockdown or exogenous expression of UBR5 altered the ubiquitination of OGA. The cells in each group were treated with the proteasomal inhibitor MG132. Cell lysates were prepared and subjected to immunoprecipitation with anti-OGA antibody. The level of ubiquitin-attached OGA was detected by western blotting with anti-ubiquitin antibody. **H** Ubiquitination of wild-type OGA or the K-to-R mutant (mutations in all Lys sites of the OGA gene) in pancreatic cancer cells. **I** Measurement of OGA ubiquitination type in pancreatic cancer cells. **J**, **K** AsPC-1-G/R and PANC-1-G/R cells were transfected with plasmid encoding HA-OGA either with or without the flag-UBR5 plasmid. Then, the cells were subjected to cycloheximide (CHX) (20 μmol/L) exposure at the indicated times, and the degradation of OGA was detected with anti-HA antibody. **L**, **M** AsPC-1-G/R and PANC-1-G/R cells transduced with shUBR5 were treated with MG132. Cells were collected at 6 h and immunoblotted with the antibodies indicated.

Next, we evaluated the mechanism underlying UBR5 modulation by OGA. UBR5 acts as an E3 ubiquitin-protein ligase that interacts with various substrates to promote their degradation [23]. Notably, OGA and UBR5 interacted as shown by co-immunoprecipitation(Fig. 6A, B). The co-localisation of UBR5/OGA in GEM-resistant PC cells was further verified by confocal microscopy (Fig. 6C). Additional evidence of the relationship between the two proteins was provided by docking analysis, which revealed binding contacts between OGA and UBR5 (Fig. 6D). These results show that UBR5 directly binds OGA in GEM-resistant PC cells. According to previous research, OGA deteriorates through the ubiquitin-proteasome system (UPS) [29]. Treatment of PC cells with MG132, a proteasome inhibitor, resulted in a huge increase in endogenous OGA protein levels (Fig. 6E), indicating that the UPS also breaks down OGA in PC cells. We examined the possibility of whether UBR5 directly mediates OGA ubiquitination. Notably, OGA polyubiquitination was higher when UBR5 was expressed ectopically than when UBR5 was knocked down (Fig. 6F, G). In addition, the findings displayed that OGA polyubiquitination was eliminated by mutations at every Lys site (Fig. 6H). As predicted, the K63R mutation in ubiquitin had no impact, whereas the Lys48 mutation virtually eliminated UBR5-mediated OGA ubiquitination (Fig. 6I). A degradation dynamics experiment revealed that the half-life of exogenously expressed OGA was considerably higher in PC cells overexpressing UBR5 than that in control cells, which was consistent with the ubiquitination (Fig. 6J, K). Furthermore, our findings demonstrated that there was no change in OGA expression following UBR5 dysregulation by MG132 (Fig. 6L, M). According to these findings, OGA is polyubiquitinated via a Lys48-dependent linkage by UBR5, which causes the proteasome degradation of OGA.

### Targeting UBR5 reverses GEM resistance in PC in vitro and in vivo

**Fig. 7:**
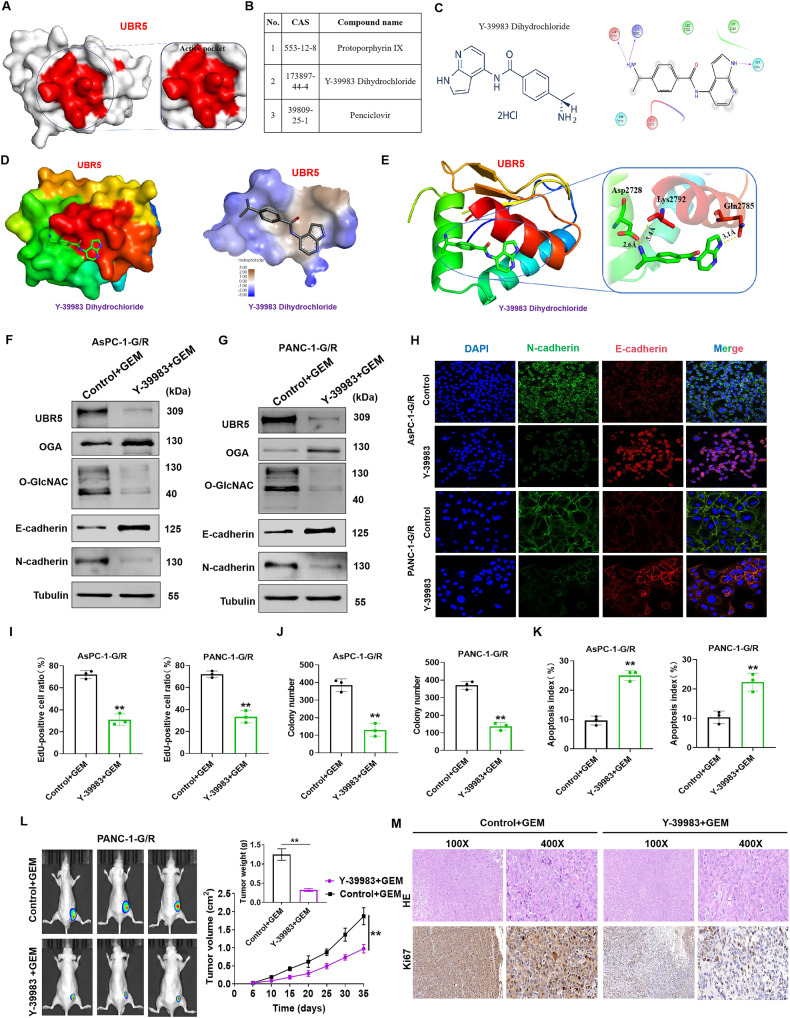
Targeting UBR5 enhances gemcitabine sensitivity in pancreatic cancer cells in vivo and in vitro. **A** UBR5 protein active pocket. **B** Drug target analyses revealed that the molecule compounds bind to the active pocket of UBR5. **C**, Chemical structure of Y-39983 dihydrochloride. **D**, **E** Protein structure and drug target analyses revealed that the Y-39983 dihydrochloride binds to the active pocket of UBR5. **F**, **G** Western blot analysis of UBR5, OGA, E-cadherin, N-cadherin expression, and O-GlcNAcylation levels in AsPC-1-G/R and PANC-1-G/R cells treated with Y-39983 dihydrochloride (5 μM). **H** Immunofluorescence analysis was used to observe the expression of EMT-related proteins (E-cadherin and N-cadherin) in AsPC-1-G/R and PANC-1-G/R cells treated with small molecule compound Y-39983 dihydrochloride (5 μM). **I** Cell viability was detected using an EdU assay of AsPC-1-G/R and PANC-1-G/R cells treated with Y-39983 dihydrochloride (5 μM, **P < 0.01). **J** Cell viability was detected using a colony formation assay in AsPC-1-G/R and PANC-1-G/R cells treated with Y-39983 dihydrochloride (5 μM,**P < 0.01). **K** The TUNEL assay to examine apoptosis in AsPC-1-G/R and PANC-1-G/R cells treated with Y-39983 dihydrochloride (5 μM,**P < 0.01). **L** Nude mice injected with luciferase-expressing AsPC-1-G/R and PANC-1-G/R cells were treated with gemcitabine alone or combined with Y-39983 dihydrochloride (100 μl of 10 μM) intraperitoneally; subcutaneous xenografts were then assessed by an IVIS imaging system (*n* = 3). The weights and volumes of subcutaneous tumours were measured. **M** Representative H&E and immunohistochemistry staining of Ki67 in tumour tissues isolated from different nude mice groups.

Our results confirmed that UBR5 expression in GEM-resistant PC cells increased, which induced GEM resistance in PC by inducing O-GlcNAcylation-mediated EMT via OGA. Therefore, we then aimed to identify a drug that targets UBR5 and inhibits O-GlcNAcylation-mediated EMT, thereby increasing the sensitivity of PC cells to GEM. The FDA drug library was screened for UBR5 inhibitors. Protein structure and drug target analyses revealed that three drugs bind to the active pocket of UBR5 (Fig. 7A–E). Y-39983 dihydrochloride caused the most significant decrease in UBR5 expression (Supplementary Fig. 5). Furthermore, the effectiveness of GEM treatment alone or in combination with Y-39983 dihydrochloride on UBR5, OGA, and EMT markers and the levels of O-GlcNAcylation were investigated by immunofluorescence and western blotting. UBR5, N-cadherin, and O-GlcNAcylation levels were downregulated, and OGA and E-cadherin were upregulated in the combination treatment group compared with those in the GEM-treated group (Fig. 7F–H). Colony formation and EdU assays were performed to assess cell proliferation and viability. Our results showed that Y-39983 dihydrochloride enhanced the inhibitory effects of GEM on AsPC-1-G/R and PANC-1-G/R cell proliferation (Fig. 7I, J). We then performed TUNEL assays to assess the effect of Y-39983 dihydrochloride on the sensitivity of AsPC-1-G/R and PANC-1-G/R to GEM. Y-39983 dihydrochloride increased the apoptotic rate in AsPC-1-G/R and PANC-1-G/R cells in response to GEM (Fig. 7K). These findings suggest that Y-39983 dihydrochloride increases the sensitivity of PC cells to GEM in vitro. To further determine the clinical significance of Y-39983 dihydrochloride in mitigating GEM resistance in PC, the effects of GEM treatment alone or in combination with Y-39983 dihydrochloride were examined in subcutaneous tumour-bearing nude mice. Figure 7L shows that the combination of GEM and Y-39983 dihydrochloride decreased tumour weight and volume. IHC analysis revealed that UBR5 and Ki-67 expression in tumours treated with the combination was lower than that in tumours treated with GEM alone (Fig. 7M). These findings demonstrate that Y-39983 dihydrochloride reverses GEM resistance in PC by inhibiting UBR5 in vivo and in vitro.

## Discussion

PC has the third-highest mortality rate of all malignant tumours of the digestive tract [30]. Nearly 85% of individuals are diagnosed an advanced stage, with a surgical resection rate of only 10–15% and a 5-year survival rate of < 9% [4]. Chemotherapy remains one of the most important means of adjuvant therapies for PC. Since 1997, the FDA has made GEM the first-line drug for treating PC, and it is now the most effective drug for treating progressive PC. However, due to the prevalence of drug resistance, recent investigations have demonstrated that GEM treatment does not improve the prognosis of patients with PC [5]. Consequently, PC detection and treatment remains extremely difficult, and it is crucial to investigate the molecular mechanisms underlying GEM resistance thoroughly and enhance the efficacy of chemotherapy for better therapeutic outcomes. Our study revealed that UBR5 is an oncogene in PC, and that GEM-resistant PC may benefit from targeting UBR5 for therapeutics.

UBR5 is an E3 ubiquitin ligase that mediates various fundamental biological processes [17]. Tumour cell invasion, metastasis, and proliferation are all aided by UBR5 overexpression [19, 23, 24]. Numerous studies have revealed a significant association between UBR5 overexpression and poor prognosis in various malignancies [17, 19, 24]. Our earlier study confirmed that UBR5 is elevated in PC tissues and is linked to disease progression [23]. According to a recent study, UBR5 is linked to drug resistance. O’ Brien et al. reported that UBR5 is a poor prognostic factor in ovarian cancer and regulates cisplatin resistance in vitro [24]. Yang et al. also demonstrated that in oestrogen receptor (ERa)+ breast cancer, UBR5 overexpression is associated with a worse prognosis and tamoxifen resistance [31]. Bian et al. demonstrated that UBR5 overexpression leads to adriamycin resistance in prostate cancer cells [32]. However, the molecular mechanisms and specific functions of UBR5 in GEM-resistant PC remain unknown. Here. we showed that UBR5 expression was remarkably elevated in drug-resistant PC cells and in GEM-resistant PC. Moreover, GEM sensitivity of PC cells was enhanced by UBR5 knockdown, both in vivo and in vitro. Furthermore, studies have demonstrated that EMT is essential for chemotherapeutic resistance in cancer [33]. Further investigation revealed that reducing UBR5 expression inhibited EMT and elevated the sensitivity of GEM-resistant PC cells; however, incorporating EMT activators prevented this process. In addition, EMT involves the metastasis of the tumor and the tumor microenvironment (TEM). In this study, we found that UBR5 affects GEM resistance in PC cells by regulating EMT. Thus, we believe that EMT regulation by UBR5 may also be involved in the metastasis of PC cells and the TME. To the best of our knowledge, this is the first study to demonstrate that UBR5 confers GEM resistance in PC cells via EMT. These results have significant implications for understanding the impact of UBR5 on GEM resistance in PC and demonstrate that UBR5 leads to GEM resistance in PC by promoting EMT.

Several intermediate metabolites such as UDP-GlcNAc, methylation, and acetylation act as direct substrates for the post-translational modification of functional proteins and actively modulate their activity, stability, and cellular events [34]. They are also affected by the metabolic adaptation of cancer cells. The process of O-GlcNAcylation, which is dynamically catalysed by OGT/OGA, involves attaching an O-linked-β-N-acetylglucosamine to the hydroxyl groups of threonine or serine [9]. O-GlcNAcylation plays a significant role in the progression of malignant tumours, and the resistance of tumour cells [13, 14]. For instance, O-GlcNAcylation, as revealed by Zhu et al. stimulates the formation of pancreatic tumours by modulating malate dehydrogenase 1 (MDH1) [35]. Yang et al. demonstrated that reducing protein O-GlcNAcylation levels significantly increases TRAIL sensitivity in PC [36]. Huat et al. demonstrated that KIAA1199 promotes oxaliplatin resistance in colorectal cancer using protein O-GlcNAcylation-mediated EMT [37]. Here, we confirmed that O-GlcNAcylation levels were increased in GEM-resistant PC cell lines and that UBR5 knockdown significantly decreased O-GlcNAcylation levels. Furthermore, in UBR5-knockdown GEM-resistant PC cells, O-GlcNAcylation slowed the loss of N-cadherin expression, but the reduction of UBR5 lowered O-GlcNAcylation levels. Our findings suggest that, following GEM treatment, apoptosis in shUBR5-transfected cells was reduced by elevating O-GlcNAcylation levels. These results indicate that O-GlcNAcylation levels are critical for UBR5 to promote GEM resistance and EMT in GEM-resistant PC cells.

OGA and OGT are primary regulators of O-GlcNAcylation [13]. Here, we report on a unique mechanism by which UBR5 promotes the degradation of ubiquitinated OGA to modulate O-GlcNAcylation. The expression of OGA was increased in GEM-resistant PC cells after the UBR5 knockdown, but OGT protein, OGA, and *OGT* mRNA were unchanged. Furthermore, our findings suggest that OGA expression was evidently decreased in the tissues of patients with GEM-resistant PC and in drug-resistant PC cells. UBR5 regulated O-GlcNAcylation-induced PC EMT and GEM resistance through an OGA-dependent mechanism. Finally, we closely examined this process to determine how UBR5 modulates OGA. OGA degradation mediated by the ubiquitin-proteasome is a key mechanism for modulating OGA levels. Lin et al. reported that N-acetyltransferase 10 (NAT10) might regulate OGA stability and expression by suppressing OGA degradation [29]. However, the potential E3 ubiquitin ligase activity of OGA has not yet been reported. These data indicate for the first time that UBR5 may act as an E3 ubiquitin ligase for OGA and that it engages in the OGA degradation process. Furthermore, docking analysis, confocal microscopy, and co-immunoprecipitation demonstrated that UBR5 directly binds to OGA in GEM-resistant PC cells. Moreover, UBR5 overexpression significantly increased OGA polyubiquitination, whereas UBR5 knockdown decreased OGA polyubiquitination. In addition, His-OGA ubiquitination was observed in vitro in the presence of E1, E2 (UBCH5c), ubiquitin, or UBR5. Furthermore, OGA is degraded in the proteasome by Lys48-linked poly-ubiquitination, which is mediated by UBR5.

This study comprehensively analysed the effects of an FDA drug, Y-39983 dihydrochloride, on PC and suggested that it could be further evaluated in clinical trials as a first- or second-line therapy in combination with GEM. Numerous studies have confirmed that Y-39983 dihydrochloride is a selective rho-associated protein kinase inhibitor that plays an important role in inhibiting malignant tumour progression [38–40]. Here, our findings showed that the Y-39983 dihydrochloride binds to the active pocket of UBR5. UBR5, N-cadherin, and O-GlcNAcylation levels were downregulated, and OGA and E-cadherin were upregulated in the combination treatment group compared with the GEM-treated group. Furthermore, Y-39983 dihydrochloride enhanced GEM efficacy against GEM-resistant PC cells. Moreover, Y-39983 dihydrochloride increased the sensitivity of PC cells to GEM in vitro. The combination of GEM and Y-39983 dihydrochloride exerted growth-inhibitory effects on tumours in immunodeficient mice. Thus, targeting UBR5 with this combination is a potential novel therapeutic strategy to improve treatment outcomes.

**Fig. 8:**
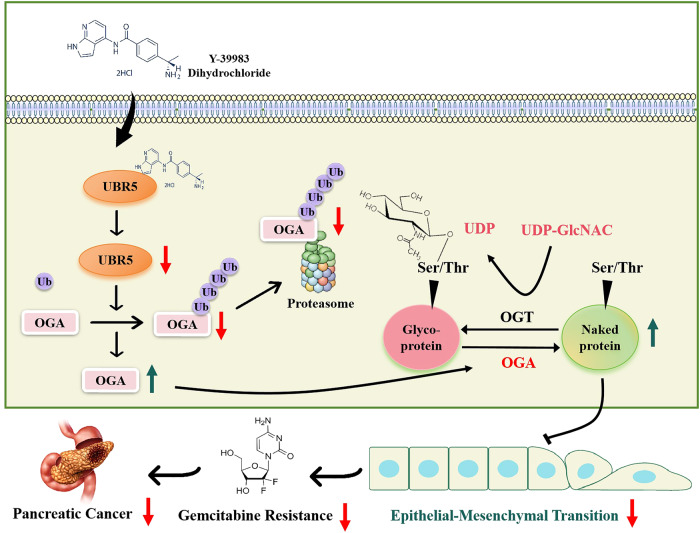
Model summarising the role of UBR5 in gemcitabine-resistant pancreatic cancer. The proposed model suggests that the E3 ubiquitin ligase UBR5 promotes EMT by facilitating the degradation of OGA via the ubiquitin proteasome, which, in turn, increases O-GlcNAc glycosylation levels, ultimately leading to gemcitabine resistance in pancreatic cancer cells. This study extensively characterised the inhibitory effect of Y-39983 dihydrochloride on gemcitabine resistance in pancreatic cancer using preclinical models.

In summary, as the first reported E3 ubiquitin ligase for OGA, UBR5 promotes EMT by facilitating OGA degradation via the ubiquitin proteasome. This increases O-GlcNAc glycosylation levels, ultimately leading to GEM resistance in PC cells. Thus, UBR5 is a promising therapeutic target for the GEM-resistant PC. Moreover, our findings support the need for further clinical assessment by combining Y-39983 dihydrochloride and GEM to treat patients with PC (Fig. 8). Additional predictors of therapeutic efficacy in these clinical trials may contribute to refining the subgroup of patients who are most likely to benefit from this combination treatment. Thus, the optimal risk-benefit ratio can be achieved for each patient, and treatment efficacy can be optimised.

## Materials and methods

### Patients and clinical samples

Clinical specimens were collected at the Second Affiliated Hospital of Nanchang University, China. Thirty patients who underwent GEM neoadjuvant chemotherapy and surgery were selected according to the Chinese Society of Clinical Oncology (CSCO) guidelines for PC diagnosis and treatment. Twelve patients with PC responded well to GEM therapy, whereas the remaining 18 exhibited resistance. Biopsies of individuals receiving palliative care or surgical resection yielded tissues which were stored at −80 °C. The clinical diagnosis of PC was based on the original histopathology. With informed consent from the patients and their families, the Medical Research Ethics Committee of the Second Affiliated Hospital of Nanchang University authorised all specimens used in this investigation.

### Immunohistochemistry (IHC)

Embedded tissue wax blocks were sliced, dewaxed, washed, and hydrated in xylene, ethanol, and phosphate-buffered saline (PBS). Sodium citrate was used for antigen repair, and hydrogen peroxide was used to block endogenous peroxidase. Anti-OGA (Proteintech) and anti-UBR5 (abcam) antibodies were incubated with tissues overnight at 4 °C. Horseradish peroxidase (HRP)-labelled goat anti-mouse/rabbit IgG (H + L) (Cell Signalling Technology) was incorporated into paraffin sections for secondary antibody binding. Haematoxylin staining of nuclei was performed. After washing in an ethanol and xylene solution, the sample was sealed with a neutral resin. An inverted fluorescence microscope was used to assess immunohistochemical staining.

### Cell lines

GEM-resistant cell lines (AsPC-1-G/R,PANC-1-G/R,and SW1990-G/R) and pancreatic cancer (PC) cell lines (AsPC-1, PANC-1, and SW1990) were preserved by our research group. No *Mycoplasma* or fungal contamination was detected in these cell lines. AsPC-1, PANC-1, and SW1990 cells were cultured in Roswell Park Memorial Institute (RPMI)-1640 and DMEM (Gibco), and the drug-resistant cell lines SW1990-G/R, PANC-1-G/R, and AsPC-1-G/R were cultured at 37 °C with various concentrations of GEM in a 5% CO_2_ incubator. Transforming growth factor-β (TGF-β) at 10 ng/mL was added to induce EMT. Drug-resistant cells were grown in plain media for 48 h after normal cultivation.

### RNA sequencing (RNA-seq) analysis

RNA samples were stored at −80° and sent to Shanghai Mingcode Biotechnology Co. Ltd. for transcriptome sequencing.

### RNA extraction and quantitative reverse transcription polymerase chain reaction (qRT-PCR)

In accordance with the manufacturer’s recommendations, TRIzol reagent (Invitrogen) was used to extract total RNA from PC parent cells (PANC-1 and AsPC-1), drug-resistant tissues, and resistant cells (PANC-1-G/R and AsPC-1-G/R). Total RNA was quantified using an Evolution 350 spectrophotometer (Thermo Fisher Scientific). The PrimeScript Reverse Transcription Reagent Kit with gDNA Eraser (TaKaRa, RR047A) was used for reverse transcription. qPCR was performed using TB Green^®^Premix Ex Taq Quantitative (Tli RNaseH Plus) (TaKaRa, RR420A). For each sample, gene expression levels were normalised to those of glyceraldehyde-3-phosphate dehydrogenase (*GAPDH*) and calculated using 2^−ΔΔct^.

### Western blot

We used RIPA lysis buffer to extract total protein from PC parent cells, drug-resistant tissues, and resistant cells. The bicinchoninic acid (BCA) method was used to determine protein concentration. Subsequently, each sample was boiled for 10 min in loading buffer, separated using 6% or 8% sodium dodecyl sulphate polyacrylamide gel electrophoresis (SDS-PAGE), and transferred onto a polyvinylidene fluoride (PVDF) membrane. Blocking with 5% skim milk at room temperature (RT) for 1 h was followed by an overnight incubation at 4 °C with the primary antibody. Three 10-min washes with 1×Tris-buffered saline, 0.1% Tween^®^ 20 (TBST) were then performed followed by an overnight incubation at RT with the matching secondary antibody. After washing three times with 1×TBST, membranes were exposed to an enhanced chemiluminescence (ECL) reagent for imaging. ImageJ software was used to analyse the data.

### Plasmid and short hairpin (sh)RNA transfection

The shUBR5 plasmid was constructed by synthesising the double stranded RNA of UBR5 using shRNA from the gemma gene (Shanghai, China). Plasmids and shRNAs were transfected into PC-resistant cells using Lipofectamine 3000 transfection reagent (Invitrogen, L3000015). Finally, PC-resistant cell lines stably transfected with sh-UBR5 or sh-NC plasmids were screened using neomycin and cultured.

### Immunofluorescence

Treated cells (3 × 10^4^ cells/mL) were inoculated on confocal dishes, incubated for 24 h, then fixed with 4% paraformaldehyde for 30 min, and washed twice with PBS. After an hour at RT, samples were blocked with 5% bovine serum albumin (BSA). After discarding the waste liquid, Anti-N-cadherin (Proteintech, 1:100) and Anti-E-cadherin (Proteintech, 1:100) were added and inoculated overnight at 4 °C. Following an hour incubation with a fluorescent secondary antibody, the nucleus was stained for two minutes with 4’,6-diamidino-2-phenylindole (DAPI) after three PBS rinses. The fluorescence intensities of the cells in the control and treatment groups were examined under a fluorescence microscope after washing three times with PBS.

### 5-Ethynyl-20-deoxyuridine (EdU) incorporation assay

We inoculated 100 μL of treated cell suspensions (3 × 10^4^ cells) on 96-well plates and incubated for 24 h. Cell growth was examined under fluorescent conditions using the Cell-Light EdU Apollo488 In Vitro Kit (RiboBio, C10310-3) according to the manufacturer’s instructions.

### Co-immunoprecipitation (IP)

The cells were centrifuged at 10,000 rpm for 15 min after being lysed for half an hour with pre-cooled IP lysate. Magnetic beads (60 μL) and primary inhibitor (5 μL) were added to the supernatant, and samples were shaken at low temperature on a ROOTER overnight. The next day, the centrifuge was pre-cooled, the supernatant was discarded, and IP cracking liquid was added to clean the centrifuge three times, each time at 3000 rpm for 5 min. Supernatants were discarded and samples were boiled in 2× buffer for 10 min. Samples were stored for western blotting.

### Annexin V apoptosis assay and terminal deoxynucleotidyl transferase dUTP nick end labelling (TUNEL)

TUNEL and the Annexin V apoptosis assay were performed as previously described. Apoptosis was investigated using a TUNEL assay with the In Situ Cell Death Detection Kit (Roche, Indianapolis, IN, USA). Additionally, apoptosis was examined by flow cytometry in cells stained with fluorescein isothiocyanate and propidium iodide-labelled annexin V.

### Tumorigenicity assay

Male BALB/c nude mice (aged 4 weeks; six mice per group) were provided by Jiangsu Zhizhuo Yaokang Biotechnology Co., Ltd. Parental PANC-1 or PANC-1-GEM-R cells (2 × 10^6^ cells per cell) were transfected stably using lentiviruses with various plasmids in 100 μL Dulbecco’s Modified Eagle Medium (DMEM) and then subcutaneously implanted into the lateral thighs of the mice. The tumour volume was assessed every other week and administration began when the tumour size reached approximately 100 mm^3^, at which time the mice were randomised to receive either dimethyl sulfoxide (DMSO; intraperitoneal injection) or GEM (50 mg/Kg/two days intraperitoneally) or small molecular compounds. The animals were euthanised 35 days after cell inoculation and the tumours were isolated and weighed. The animal experiments were conducted on an animal platform at the Biomedical Testing Centre of Nanchang University. Animals were cared for and handled according to the guidelines of the Animal Platform.The animal study protocol was approved by the Laboratory Animal Ethics Committee of Nanchang University (NCUFII-2020523) for studies involving animals.

### Statistical analysis

GraphPad Prism 9.0 and SPSS 21.0 were used for all statistical analyses. Mean ± standard deviation (SD) was used to represent the data. One-way analysis of variance and Bonferroni’s multiple comparisons test were utilized for the comparison of means between more than two groups, whereas the two-tailed unpaired Student’s t-test was employed to compare means between two groups. Analysis of survival was conducted with Kaplan-Meier analysis. The log-rank test was used to compare the survival curves of the mouse models. All functional in vitro experiments are representative of a minimum of three replicates. All experiments were conducted three times. At P < 0.05, differences were considered significant.

### Supplementary information

Supplementary Figure legends

Supplementary Figure 1

Supplementary Figure 2

Supplementary Figure 3

Supplementary Figure 4

Supplementary Figure 5

Full and uncropped western blot

## Data Availability

All data in our study are available from the corresponding author upon reasonable request. All data generated or analyzed during this study are included in this published article. Additional datasets used and/or analyzed during the current study are available from the corresponding author on reasonable request.
