# Supplementary material for: E3 ubiquitin ligase UBR5 promotes gemcitabine resistance in pancreatic cancer by inducing O-GlcNAcylation-mediated EMT via destabilization of OGA
Source: Cell Death Dis. 2024 May 16;15(5):340. doi: 10.1038/s41419-024-06729-z (PMC11099055; doi:10.1038/s41419-024-06729-z)
Supplement: Supplementary file 1 — Supplementary Figure legends [file 41419_2024_6729_MOESM1_ESM.doc]

**Supplementary Figure legends**

**Supplementary Figure 1.UBR5 promotes GEM resistance by inducing EMT in ANC-1-G/R cells**

**A,** Cell viability was detected using the EdU assay in PANC-1-G/R cells subjected to the indicated treatments(**P<0.01). **B,** Cell viability was detected using the colony formation assay in PANC-1-G/R cells subjected to the indicated treatments(**P<0.01). **C-D,** The TUNEL or Flow cytometry assay to examine apoptosis in PANC-1-G/R cells subjected to the indicated treatments(*P<0.05,**P<0.01).

**Supplementary Figure 2.The O-GlcNAcylation increase attenuated the loss of N-cadherin in UBR5-knockdown PANC-1-G/R cells**

**A,** Immunofluorescence analysis was used to observe the expression of EMT-related proteins (E-cadherin and N-cadherin) in indicated treatment group.

**Supplementary Figure 3. O-GlcNAcylation is essential for UBR5 to induce gemcitabine resistance and EMT in PANC-1-G/R cells**

**A,** Western blot analysis of UBR5, E-cadherin expression, and O-GlcNAcylation levels in PANC-1-G/R subjected to the indicated treatments. **B,** Cell viability was detected using an EdU assay of PANC-1-G/R cells subjected to the indicated treatments (**P<0.01). **C,** Cell viability was detected using a colony formation assay of PANC-1-G/R cells subjected to the indicated treatments (**P<0.01,). **D,** The TUNEL assay to examine apoptosis in PANC-1-G/R cells subjected to the indicated treatments (**P<0.01).

**Supplementary Figure 4. Scatter plots of UBR5 and OGA protein expression in pancreatic cancer.**

**A,** Scatter plots revealed that UBR5 and OGA protein expression levels were negatively correlated in PC tissues (**P<0.01).

**Supplementary Figure 5. The Y-39983 Dihydrochloride caused the most significant decrease in UBR5 expression in** AsPC-1-G/R and **PANC1-G/R cells**

**A,** Western blot analysis of UBR5 levels in AsPC-1-G/R cells subjected to the indicated treatments. **B,** Western blot analysis of UBR5 levels in PANC-1-G/R subjected to the indicated treatments.
