# Supplementary figures and images for: E3 ubiquitin ligase UBR5 promotes gemcitabine resistance in pancreatic cancer by inducing O-GlcNAcylation-mediated EMT via destabilization of OGA
Source: Cell Death Dis. 2024 May 16;15(5):340. doi: 10.1038/s41419-024-06729-z (PMC11099055; doi:10.1038/s41419-024-06729-z)

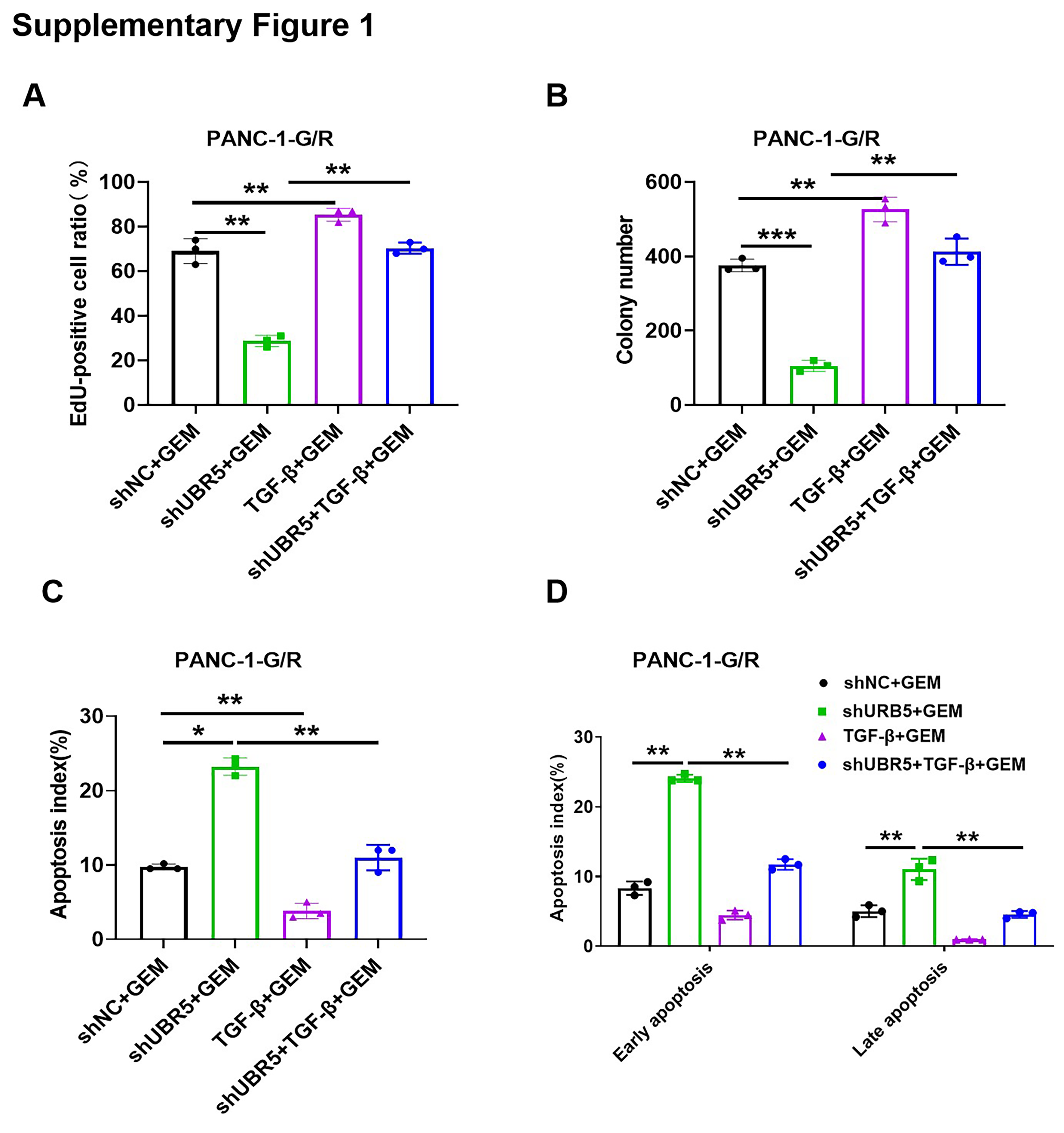

Supplement: Supplementary file 2 — Supplementary Figure 1 [file 41419_2024_6729_MOESM2_ESM.tif]

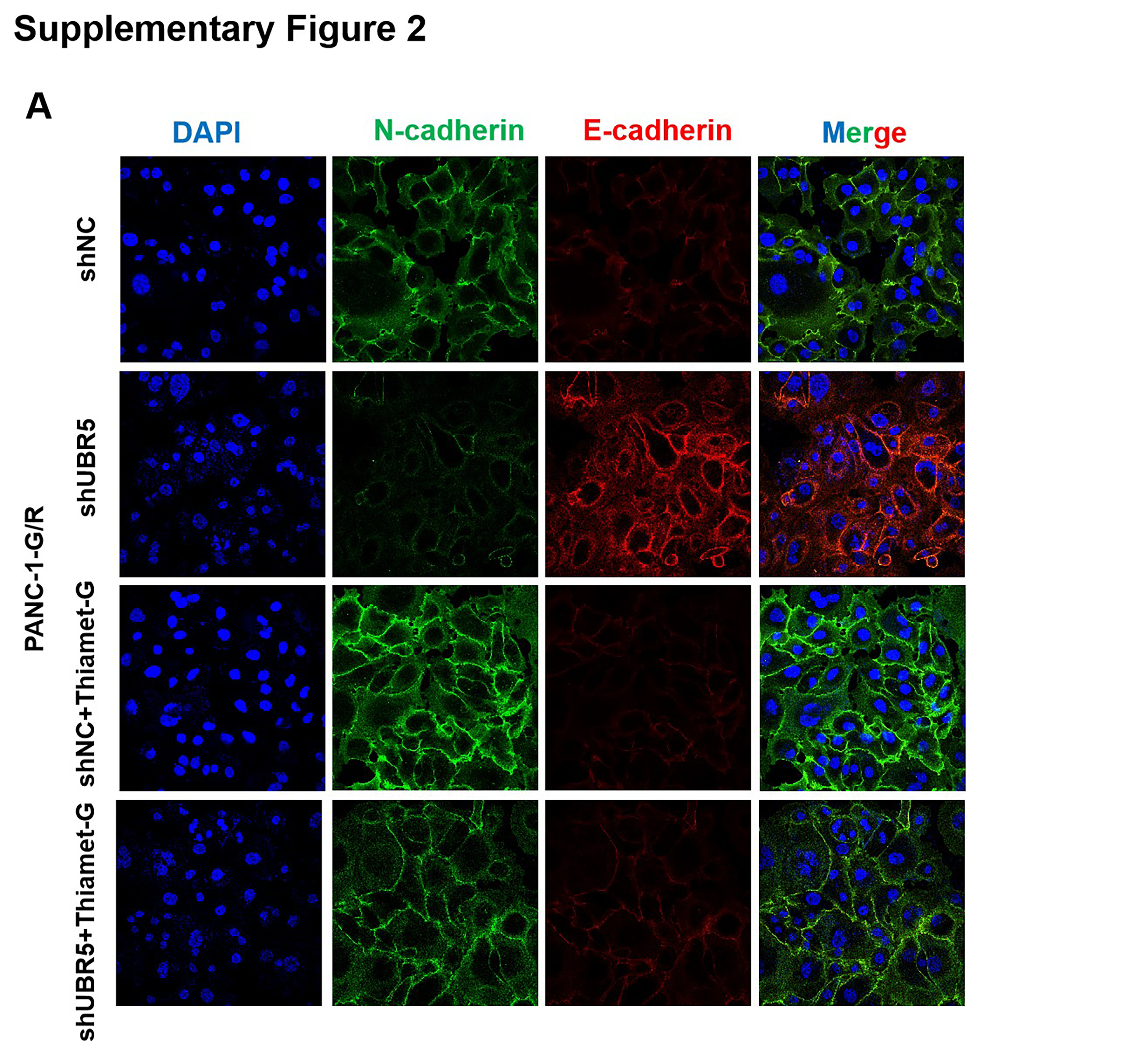

Supplement: Supplementary file 3 — Supplementary Figure 2 [file 41419_2024_6729_MOESM3_ESM.tif]

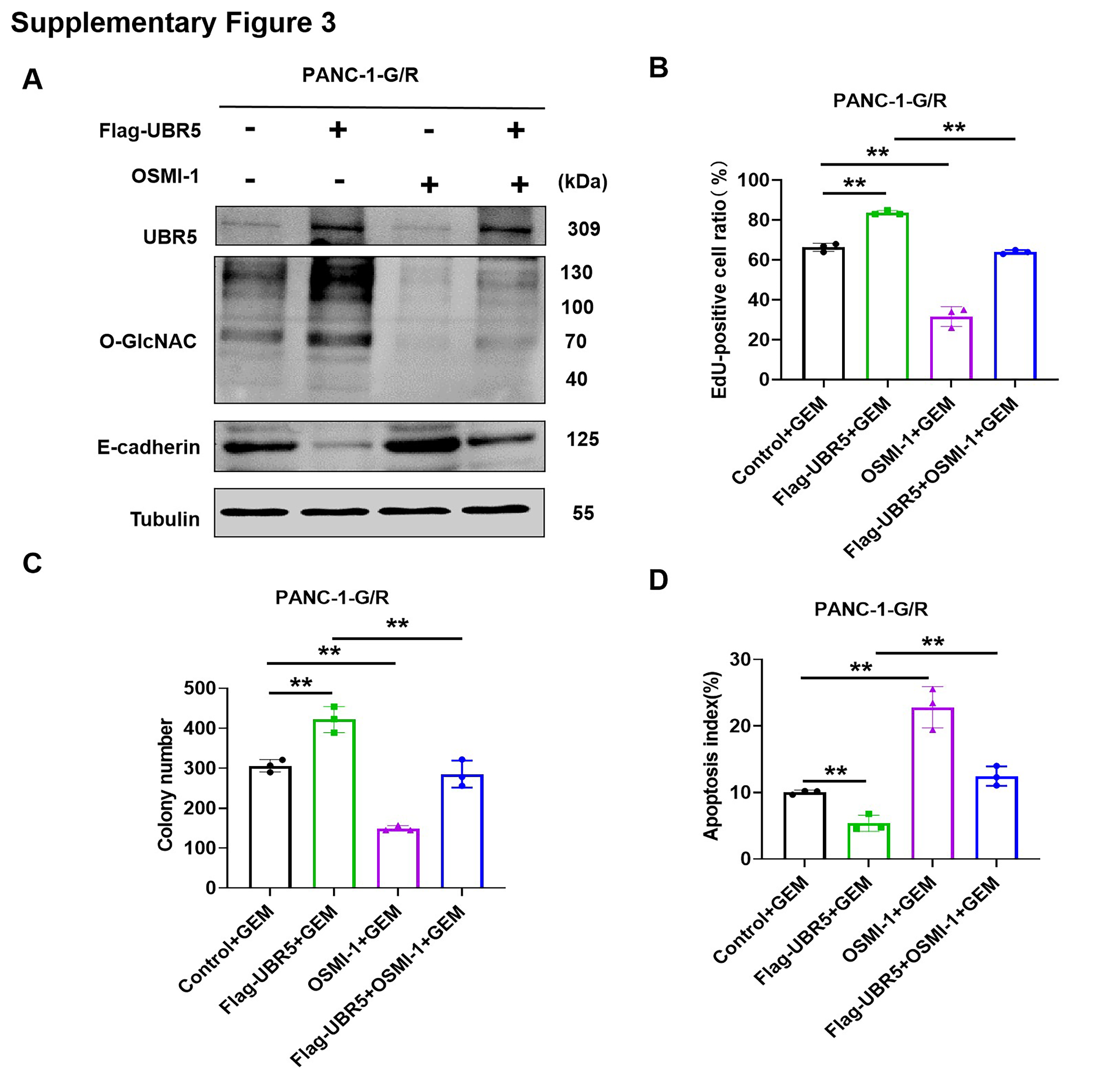

Supplement: Supplementary file 4 — Supplementary Figure 3 [file 41419_2024_6729_MOESM4_ESM.tif]

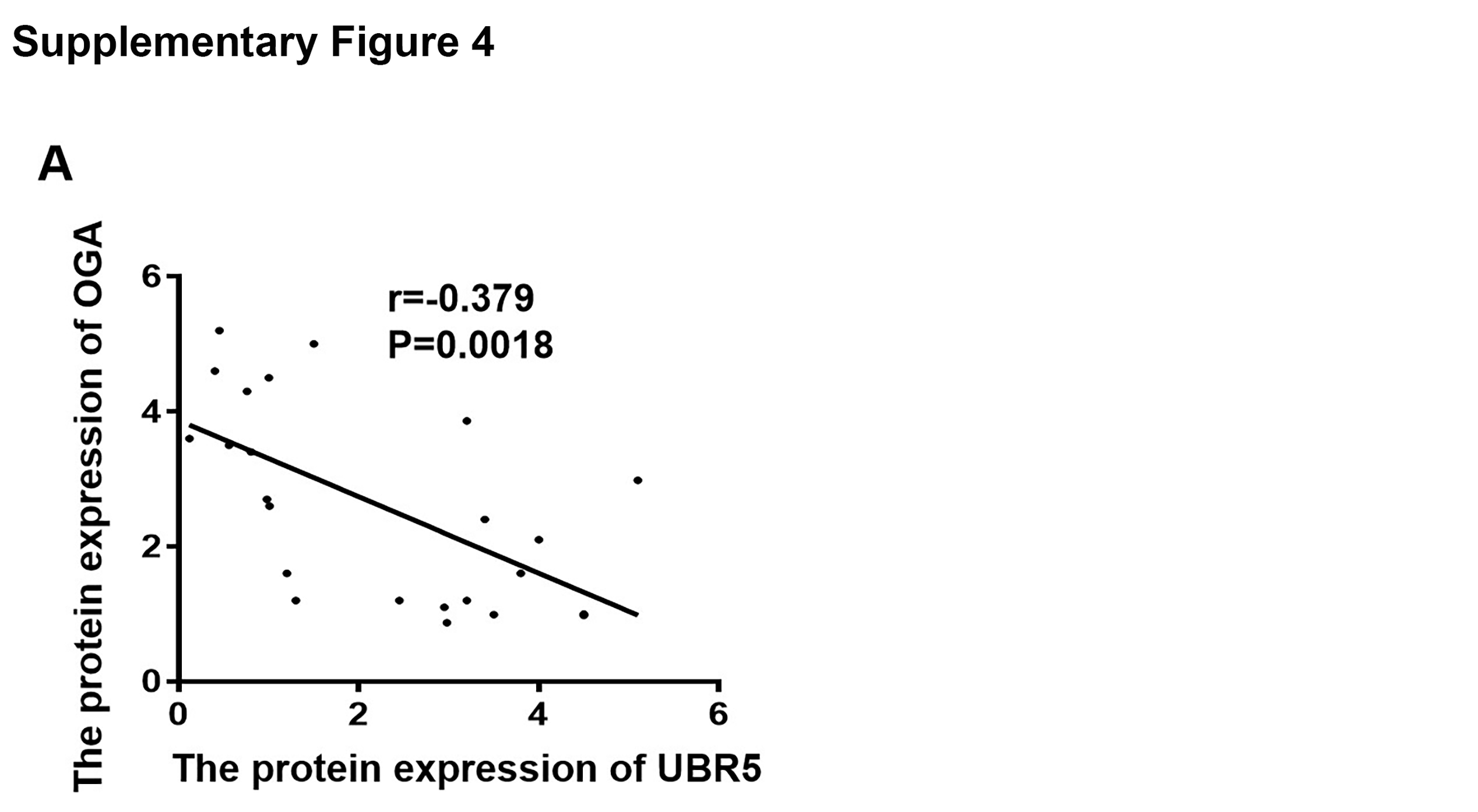

Supplement: Supplementary file 5 — Supplementary Figure 4 [file 41419_2024_6729_MOESM5_ESM.tif]

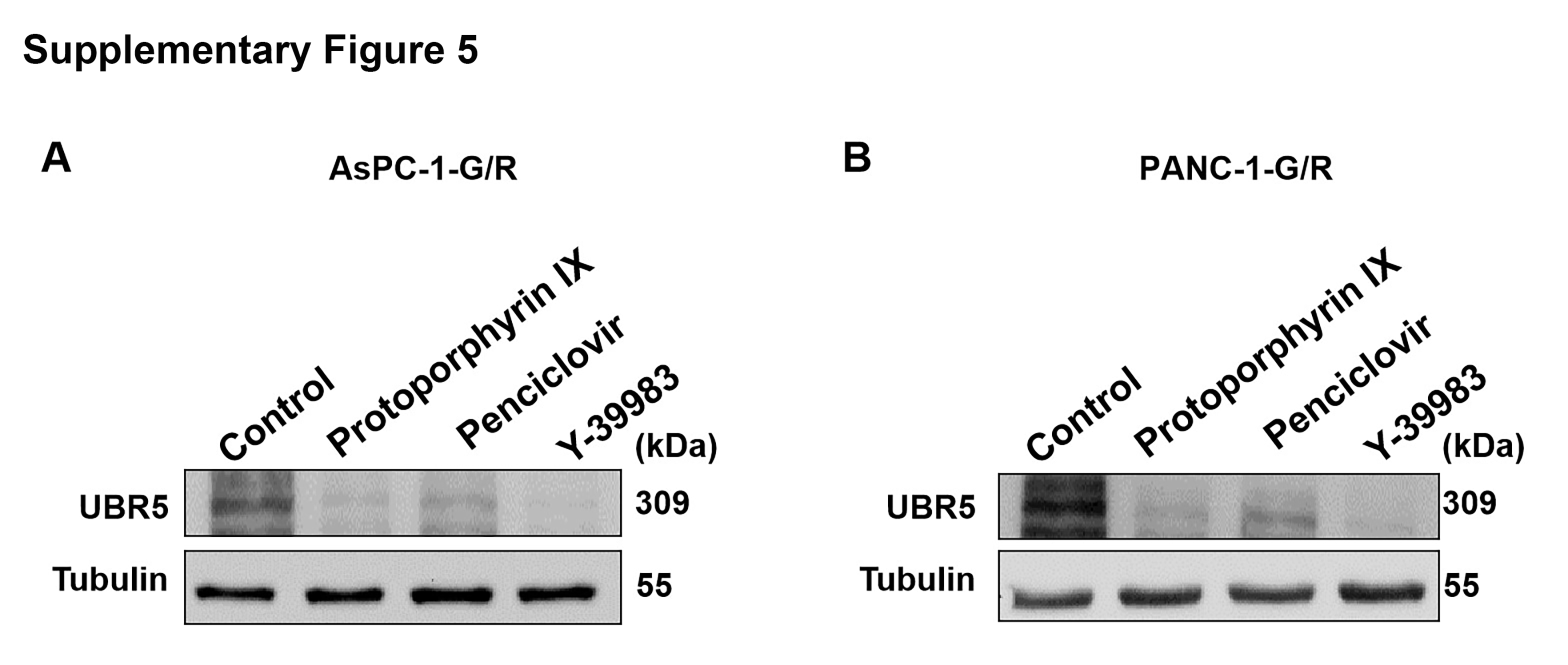

Supplement: Supplementary file 6 — Supplementary Figure 5 [file 41419_2024_6729_MOESM6_ESM.tif]
