# Supplementary material for: E3 ubiquitin ligase UBR5 promotes gemcitabine resistance in pancreatic cancer by inducing O-GlcNAcylation-mediated EMT via destabilization of OGA
Source: Cell Death Dis. 2024 May 16;15(5):340. doi: 10.1038/s41419-024-06729-z (PMC11099055; doi:10.1038/s41419-024-06729-z)

Full and uncropped western blot for Figure 1

Figure 1G

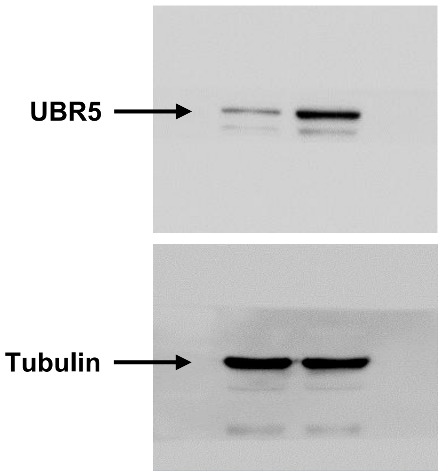

Figure 1G

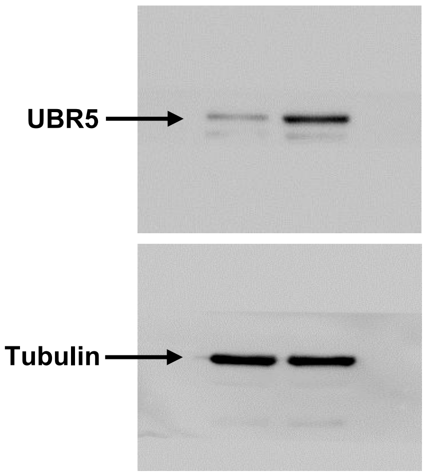

Figure 1G

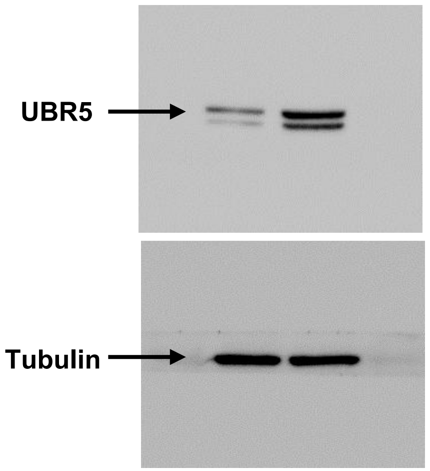

Figure 1J

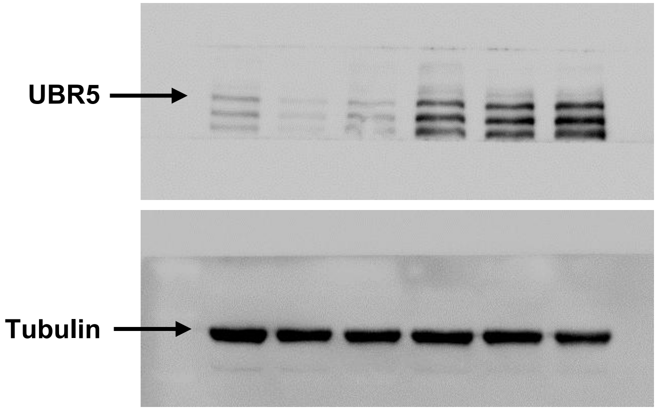

Figure 1J

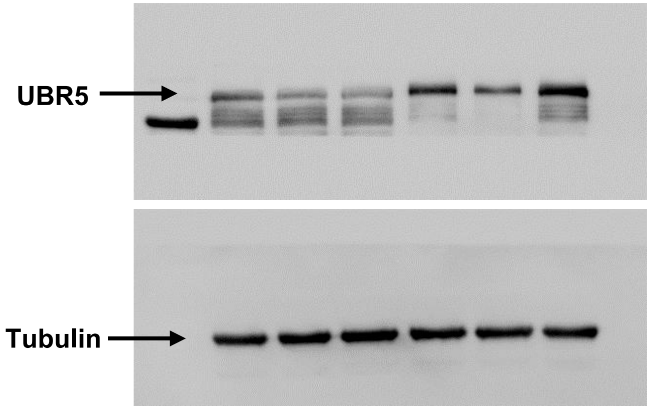

Full and uncropped western blot for Figure 2

Figure 2A

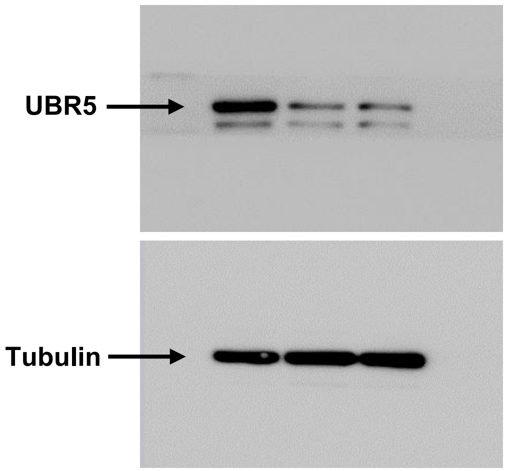

Figure 2A

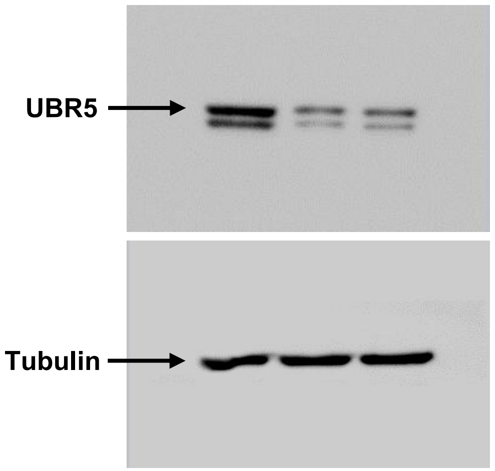

Full and uncropped western blot for Figure 3

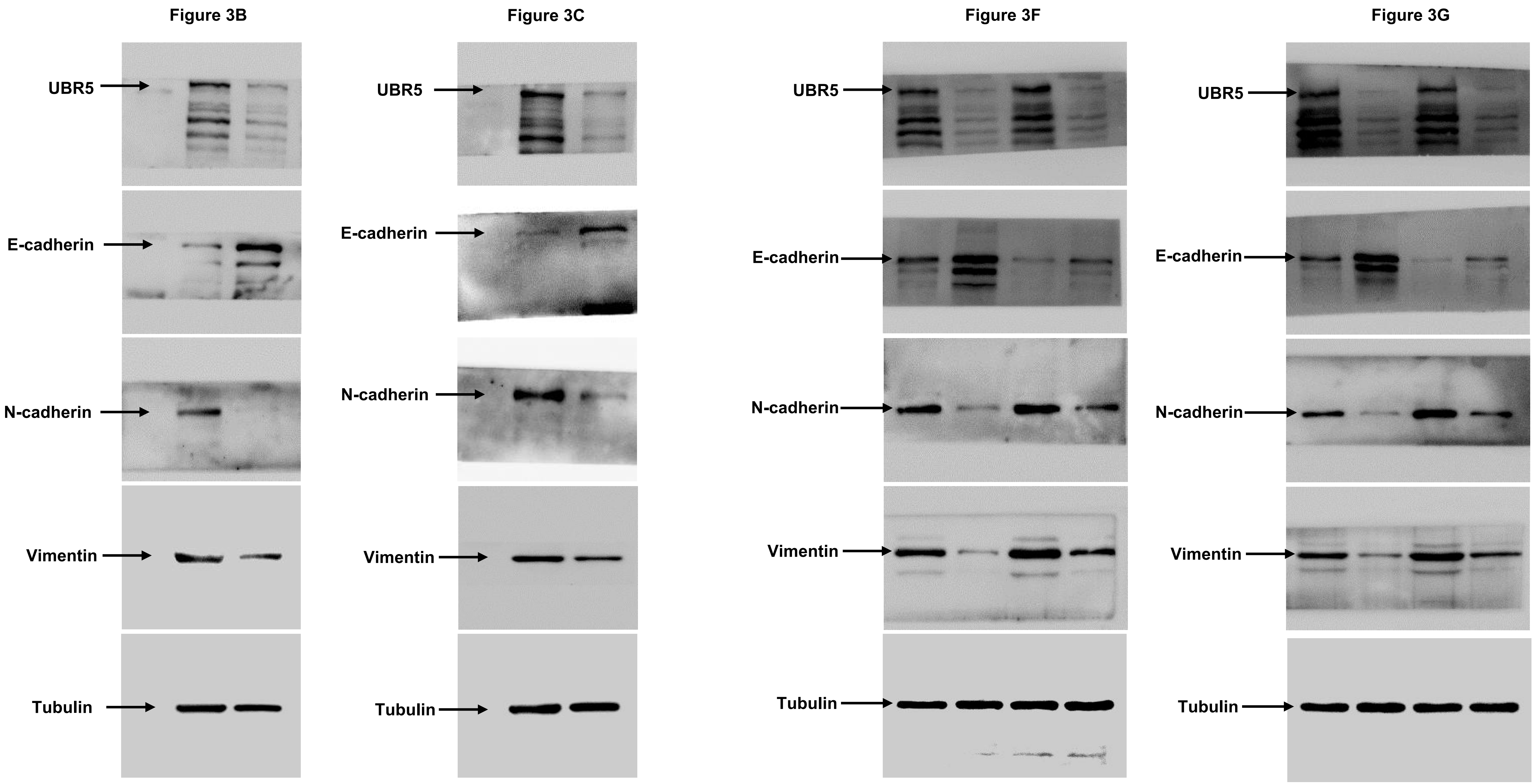

Full and uncropped western blot for Figure 4

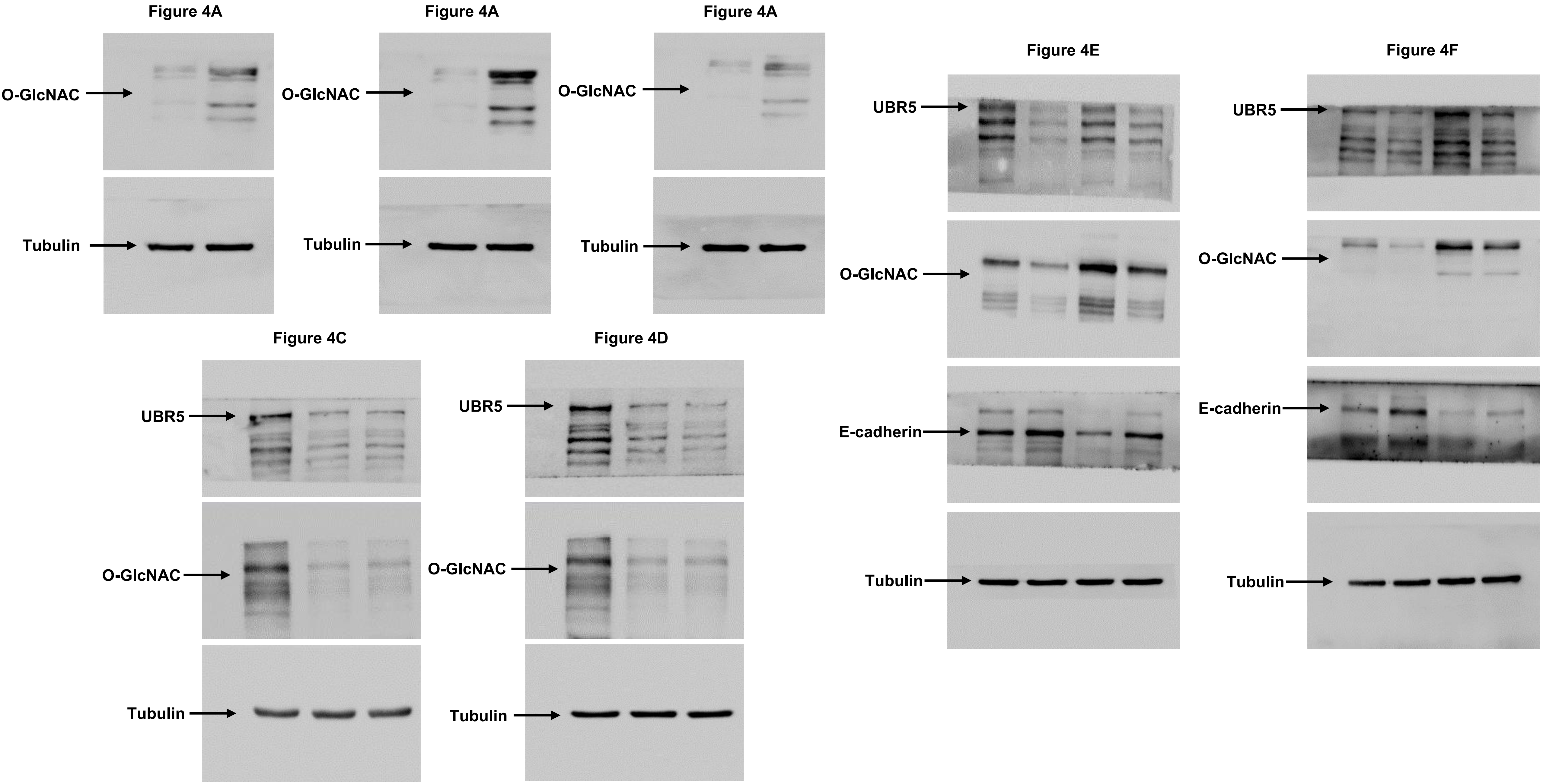

Full and uncropped western blot for Figure 5

Figure 5A

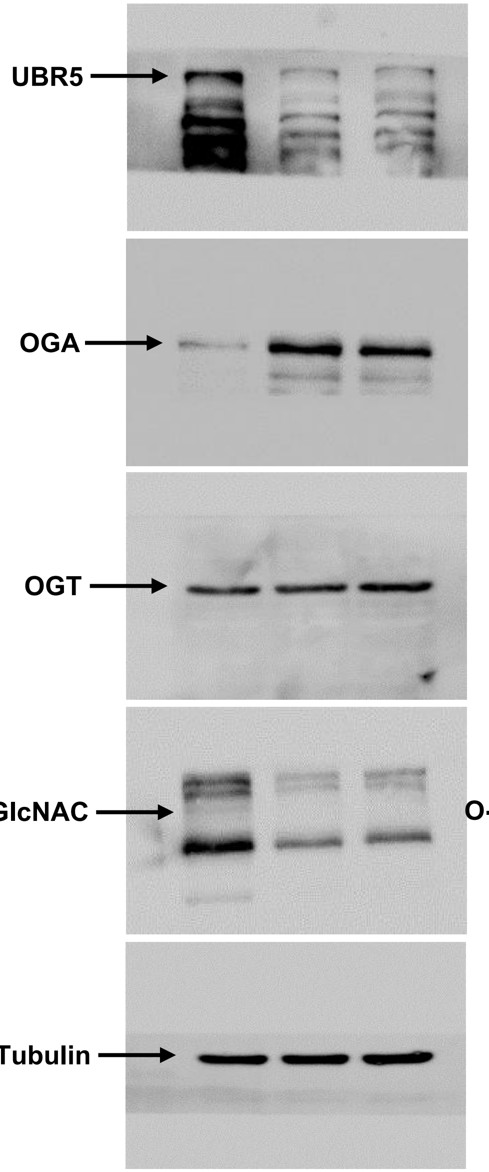

Figure 5B

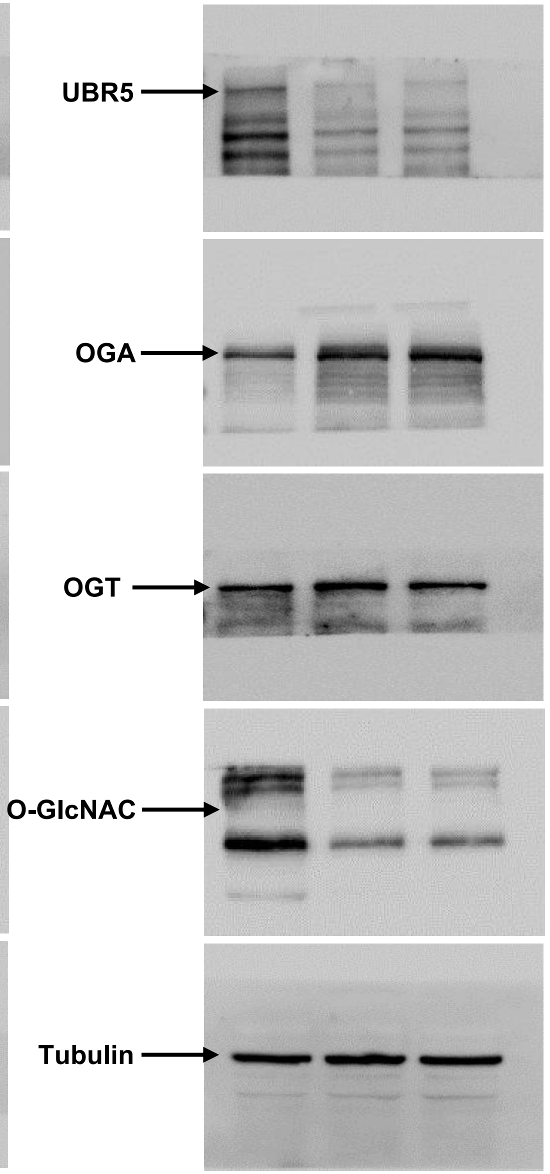

Figure 5E

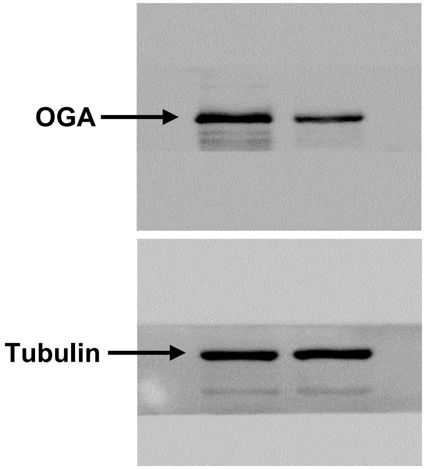

Figure 5E

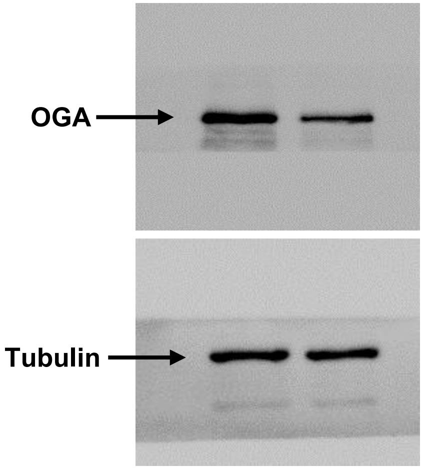

Figure 5H

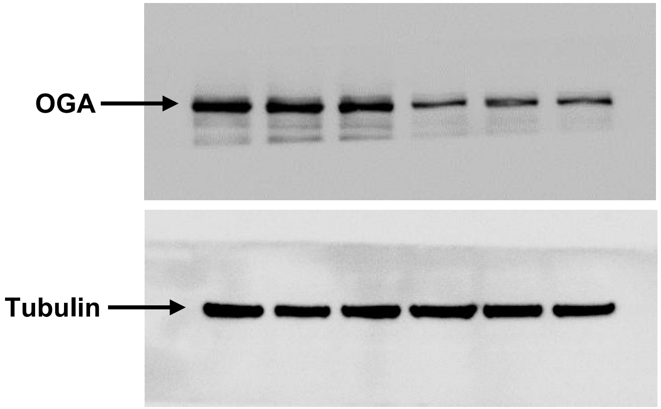

Figure 5J

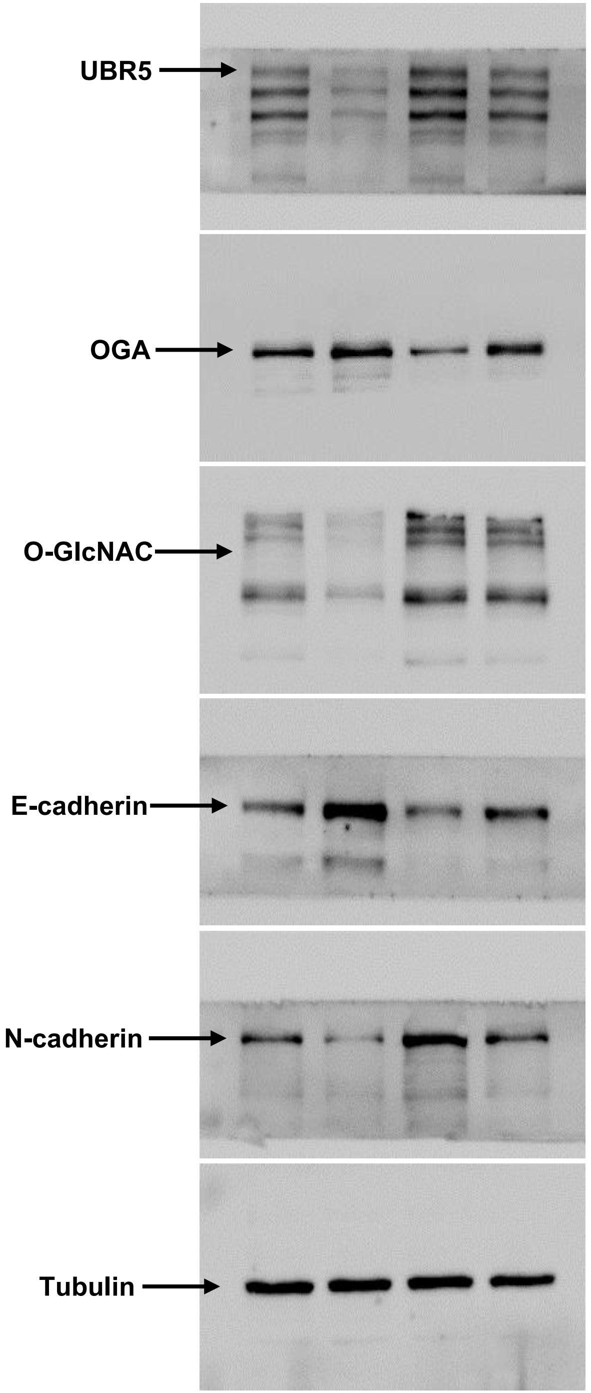

Figure 5K

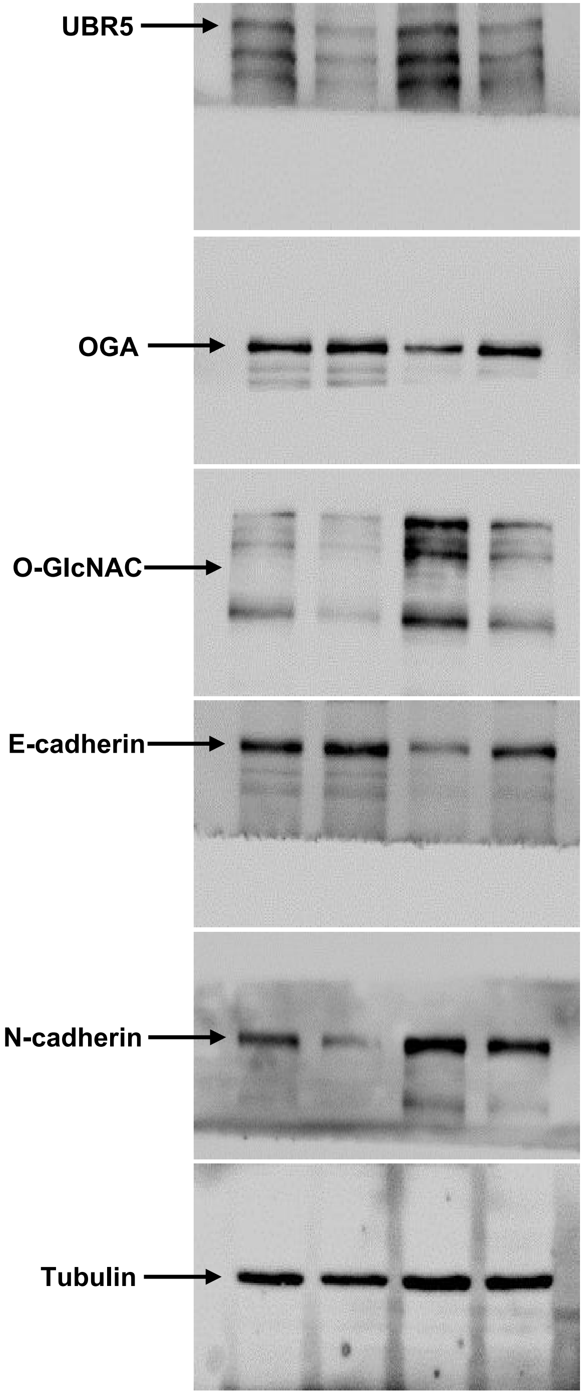

Full and uncropped western blot for Figure 6

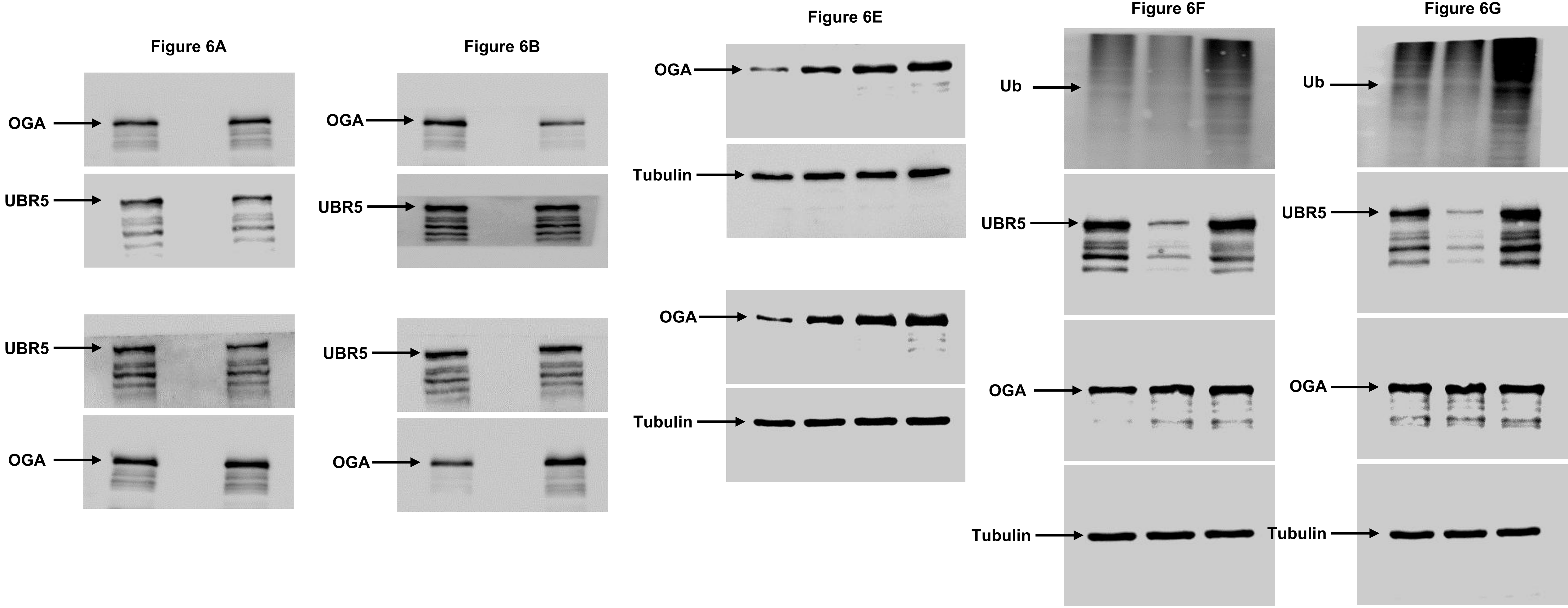

Full and uncropped western blot for Figure 6

Figure 6H

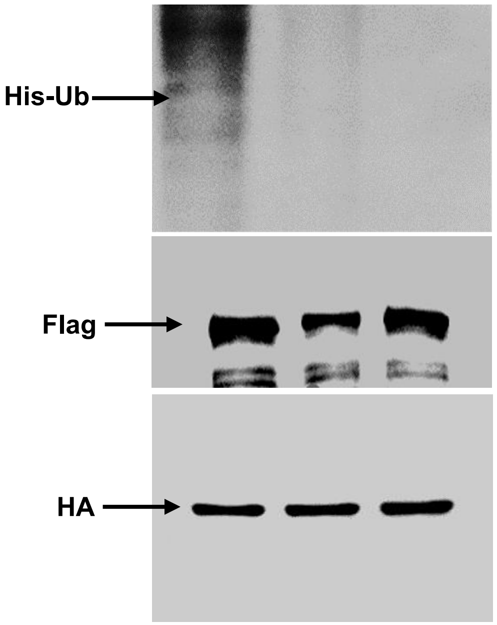

Figure 6I

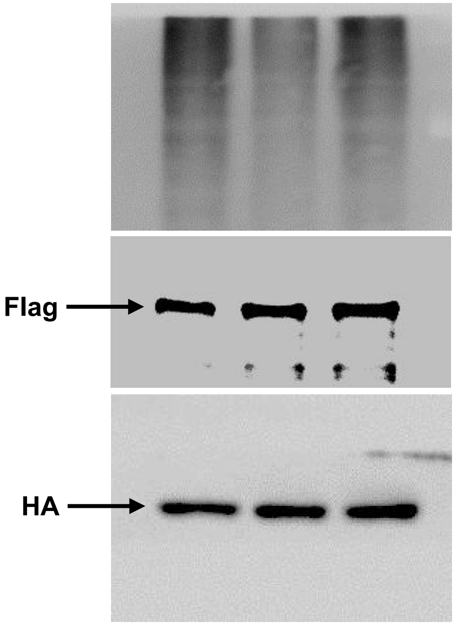

Figure 6J

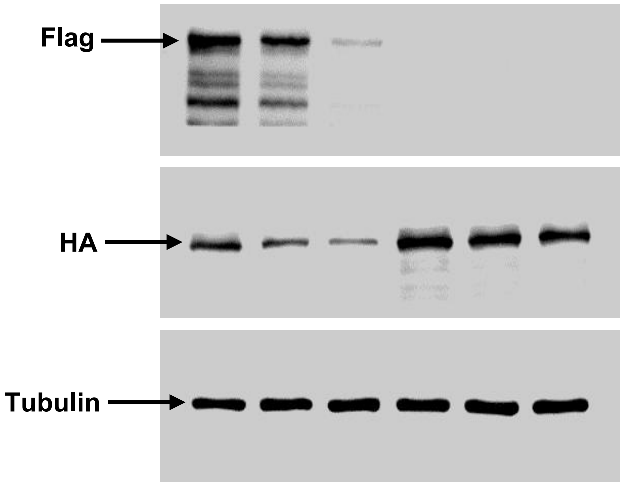

Figure 6K

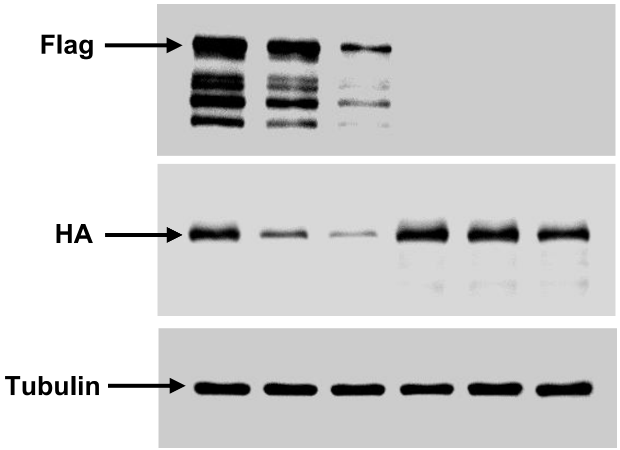

Figure 6L

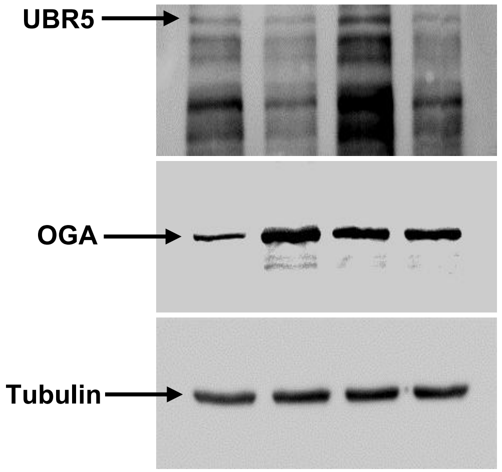

Figure 6M

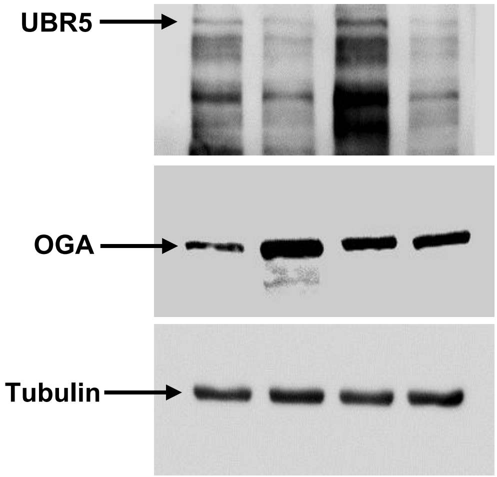

Full and uncropped western blot for Figure 7

Figure 7F

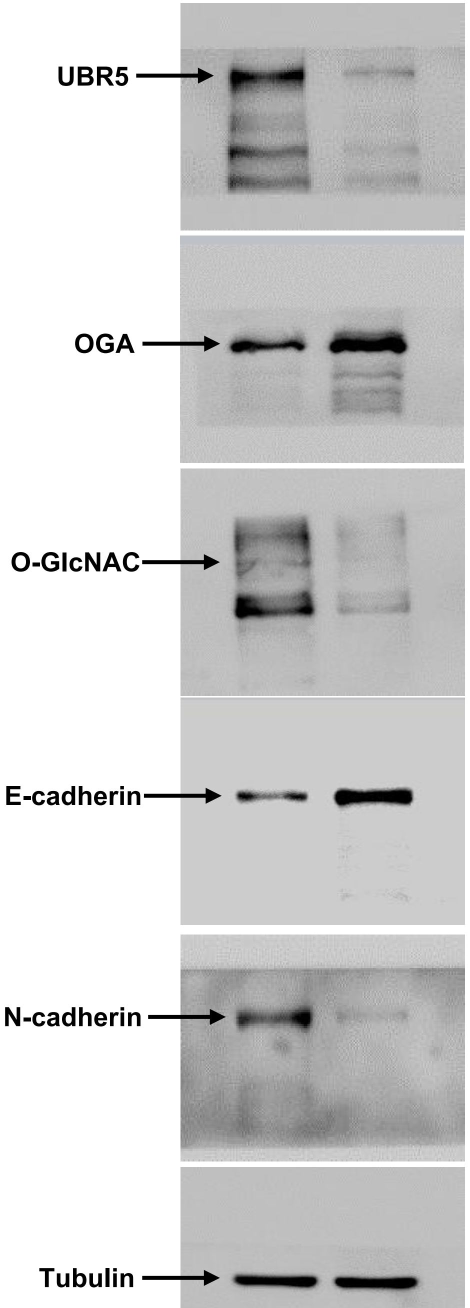

Figure 7G

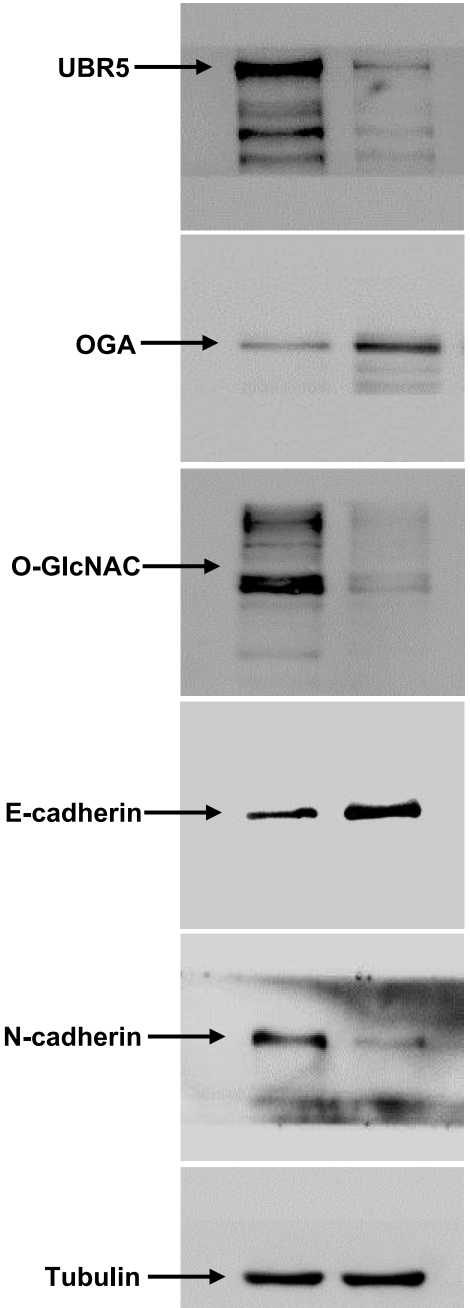

Full and uncropped western blot for Supplementary Figure 3

Supplementary Figure 3A

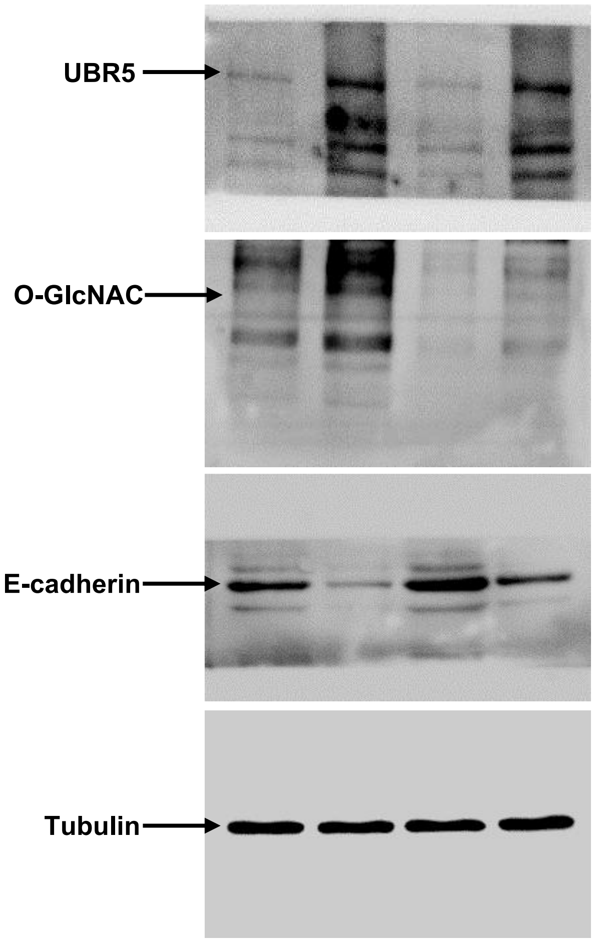

Full and uncropped western blot for Supplementary Figure 5

Supplementary Figure 5A

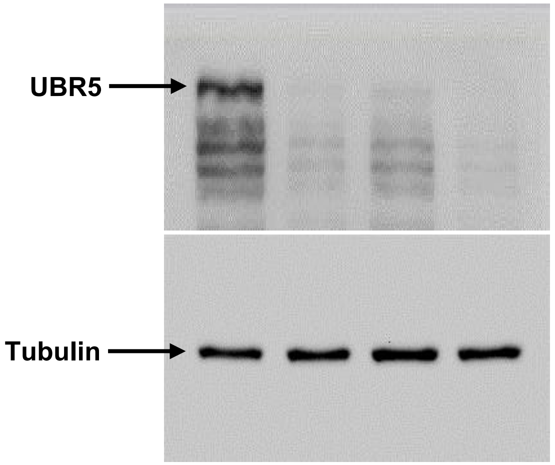

Supplementary Figure 5B

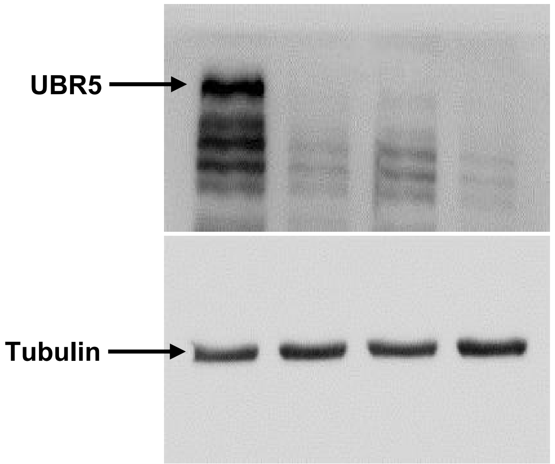

Supplement: Supplementary file 7 — Full and uncropped western blot [file 41419_2024_6729_MOESM7_ESM.pdf]
